# Religious affiliation and immunization coverage in 15 countries in Sub-Saharan Africa

**DOI:** 10.1016/j.vaccine.2019.11.024

**Published:** 2020-01-29

**Authors:** Janaína Calu Costa, Ann M. Weber, Gary L. Darmstadt, Safa Abdalla, Cesar G. Victora

**Affiliations:** a International Center for Equity in Health, Federal University of Pelotas, Rua Marechal Deodoro 1160, 3rd floor, 96020-220, Pelotas, RS, Brazil; b School of Community Health Sciences, University of Nevada, Reno, NV, United States; c Department of Pediatrics, Stanford University School of Medicine, Stanford, CA, United States

**Keywords:** Demographic surveys, Religion, Immunization, Gender bias

## Abstract

**Background:**

Although religious affiliation has been identified as a potential barrier to immunization in some African countries, there are no systematic multi-country analyses, including within-country variability, on this issue. We investigated whether immunization varied according to religious affiliation and sex of the child in sub-Saharan African (SSA) countries.

**Methods:**

We used data from 15 nationally representative surveys from 2010 to 2016. The major religious groups were described by country in terms of wealth, residence, and education. Proportions of fully immunized and unvaccinated children were stratified by country, maternal religion, and sex of the child. Poisson regression with robust variance was used to assess whether the outcomes varied according to religion, with and without adjustment for the above cited sociodemographic confounders. Interactions between child sex and religion were investigated.

**Results:**

Fifteen countries had >10% of families affiliated with Christianity and >10% affiliated with Islam, and four also had >10% practicing folk religions. In general, Christians were wealthier, more educated and more urban. Nine countries had significantly lower full immunization coverage among Muslims than Christians (pooled prevalence ratio = 0.81; 95%CI: 0.79–0.83), of which seven remained significant after adjustment for confounders (pooled ratio = 0.90; 0.87–0.92). Four countries had higher coverage among Muslims, of which two remained significant after adjustment. Regarding unvaccinated children, six countries showed higher proportions among Muslims, all of which remained significant after adjustment [crude pooled ratio = 1.83 (1.59–2.07); adjusted = 1.31 (1.14–1.48)]. Children from families practicing folk religions did not show any consistent patterns in immunization. Child sex was not consistently associated with vaccination.

**Conclusion:**

Muslim religion was associated with lower vaccine coverage in several SSA countries, both for boys and girls. The involvement of religious leaders is essential for increasing immunization coverage and supporting the *leave no one behind* agenda of the Sustainable Development Goals.

## Highlights

• We analyzed child immunization coverage by family religion in 15 sub-Saharan African countries.
• This is the first within-country study of immunization according to religion across multiple countries.
• Muslim religion was associated with lower vaccination coverage in 7 of the 15 countries.
• The observed differences remained after accounting for sociodemographic factors.
• We highlight the importance of involving religious leaders in promoting immunization.

## 1 Background

Although important progress has been made in child health and survival worldwide, significant disparities within regions and countries persist [1]. The Sustainable Development Goals (SDGs), proposed by the United Nations and formally adopted by all member states in 2015, include target 3.8 of achieving universal health coverage, and leaving no one behind [2]. Yet, over 5 million children under the age of five still die every year, many from vaccine-preventable illnesses [3]. Universal coverage of preventive interventions such as immunizations would make a major contribution to reducing the mortality of children [1]. For instance, it has been estimated that more than 17 million deaths would be avoided in 73 priority countries from 2011 to 2020 by providing vaccines to children under-five years of age [4].

In order to identify children and families who are being left behind, SDG 17.18 recommends that national statistics should be “disaggregated by income, gender, age, race, ethnicity, migratory status, disability, geographic location and other characteristics relevant in national contexts” [5]. Whereas the global child survival literature is plentiful with analyses of inequalities according to wealth, gender, and geographic location, there are fewer studies on other axes of inequalities. In particular, we identified very few multi-country studies addressing within-country disparities in child health according to religious affiliation.

Some evidence suggests that religious affiliation may affect child health. Akseer and colleagues [6] showed that Muslim-majority countries (MMCs) presented higher maternal and child mortality and lower coverage regarding several reproductive, maternal, newborn and child health interventions than countries with less than 50% of the population reporting to be Muslims. The authors attributed this finding to political instability and conflicts in MMCs, as well to low empowerment of women and girls [6]. An earlier study by Razzak and colleagues [7] found that both maternal and infant mortality rates were about twice as high in MMCs than in non-MMCs and the predictors that seemed to explain most of the differences were gross national income, literacy rate, access to clean water, and level of corruption. However, neither study evaluated within-country differences across religious groups. Moreover, some countries such as Nigeria were categorized as non-MMCs, even though Islam was practiced by a substantial proportion of the population, albeit not a clear majority, thus potentially underestimating the role of religion.

Sub-Saharan Africa (SSA) is an ideal setting for studying the association between religious affiliation and child health. First, it is the region of the world with the highest mortality rates for children, with an overall under-five mortality rate of 79 per 1000 live births in 2018 [3]. Second, it is known for high proportions of the population having religious affiliation and engagement in religious practices [8]. Third, in many African countries, the majority of the population are affiliated with one or the other of the world’s two largest religions, Christianity and Islam [9]. Also, folk religious practices are reported by about 3% of the population in SSA countries [10]. This group, however, is often omitted in surveys and in several countries respondents may have more than one affiliation and fail to report folk practices. [11].

In the present analyses, we investigated whether immunization coverage varied according to religious affiliation in SSA countries with recent survey data. We also investigated whether religion might be associated with differential coverage for boys and girls.

## 2 Methods

### 2.1 Data sources

We used publicly available datasets from Demographic and Health Surveys (DHS) and Multiple Indicator Cluster Surveys (MICS) (https://www.dhsprogram.com/, http://mics.unicef.org). Both initiatives collect nationally representative data from probability multi-stage samples of households in low- and middle-income countries (LMICs). Within selected households, all women of reproductive age (15–49 years) provided information about their households, themselves and their children. The surveys use comparable methodologies for data collection and similar questionnaires that allow comparisons across countries. [12], [13] We used the most recent data from each country carried out from 2010 onwards. Ethical clearance was the responsibility of the institutions that administered the surveys and all analyses relied on anonymized datasets.

### 2.2 Outcomes

The two outcomes under study were full immunization coverage and the proportion of unvaccinated children. Full immunization coverage was defined as the proportion of children aged 12–23 months who had received at any age the following vaccines: one dose of Bacille Calmette-Guérin (BCG), three doses of vaccine against diphtheria, pertussis, and tetanus (DPT) or tetravalent/pentavalent vaccine, three doses of polio vaccine, and one dose of measles vaccine (either as monovalent vaccine or as measles-containing vaccine combinations with other immunogens) [14]. Unvaccinated children were those in the same age range who had not received any of the four vaccines listed above. Children with missing data were treated as not having received the intervention (a conservative assumption) and we did not use imputation for missing values.

### 2.3 Main exposure

Information on religious affiliation was obtained from country-specific questions in the individual or household questionnaire, depending on the type of survey. The original variable on a woman’s religion (from DHS datasets) or religion of the head of household (from MICS) was recoded into eight groups according to Pew Forum on Religion and Public Life classification: Buddhist, Christian, Folk and traditional religions, Hindu, Jewish, Muslim, Unaffiliated, and Other [10]. The categories from the original variables allocated to each group are shown in the Supplementary Material. For the MICS databases, the affiliation was defined for the household and attributed to each woman in the household at the individual level. In the analysis, we included countries with at least 10% of the women’s sample in each group of Christians and Muslims; when a third religion accounted for 10% or more of the sample, it was also included in the analyses.

### 2.4 Explanatory variables

Multivariable analyses considered the differences between religious groups in terms of wealth, urban-rural residence and women’s education. Wealth quintiles were derived using principal component analyses of household assets and building characteristics in each country; the first quintile represents the poorest 20% of households, and the fifth quintile the wealthiest 20% in the survey sample [15], [16]. Urban and rural residence was defined by each country at the time of the survey. Women’s education was classified as none, primary, and secondary or higher.

### 2.5 Analysis

The major religious groups were described in terms of their distribution of wealth, residence, and women’s education at the country-level. We calculated point estimates, standard errors and confidence intervals for the outcomes by religious group and child sex in each country. The results are presented as ratios and Pearson’s chi-squared test was used to test differences across religious groups and sex. A series of country-specific Poisson regression models with robust variance were used to assess whether each of the two outcomes varied according to religion, with and without adjustment for wealth, residence and education [17]. Interactions between sex and religion were investigated in the regression models. Using Pearson’s coefficient of correlation, we also tested the linear association between adjusted ratios (Muslim-to-Christian prevalence) for both outcomes and the proportion of Muslim women in the national samples. All analyses accounted for the multi-stage survey design, including sampling weights and clustering, using complex survey analysis functions of Stata® SE version 15 (StataCorp LP, College Station, Texas, United States), which allow robust variance estimates [18].

## 3 Results

Of the 50 SSA countries, 24 have had a DHS or MICS since 2010. Of these, 15 (10 DHS and 5 MICS) had information on vaccine coverage and included at least 10% of the sample respondents affiliated with Christian religion, and another 10% or more with Muslim religion. The median survey year was 2014. Table 1 describes the distribution of religions by country. Christians were the largest group in nine of the 15 countries. The proportions of survey respondents who were Christian ranged from 21.2% in Sierra Leone to 86.9% in Malawi, whereas Muslims ranged from 10.9% in Liberia to 78.5% in Sierra Leone. Folk and traditional religions were included in the analysis for four countries, namely Benin (16.3%), Guinea Bissau (17.7%), Mozambique (12.2%), and Togo (14.3%).

**Table 1:** Distribution of religious groups, by country.

Country	Year	Source	Christian	Muslim	Folk	Other	Unaffiliated	Largest group
Benin	2014	MICS	**50.7%**	**26.9%**	**16.3%**	0.7%	5.4%	Christian
Burkina Faso	2010	DHS	**30.0%**	**61.8%**	7.4%	0.0%	0.9%	Muslim
Cameroon	2014	MICS	**69.8%**	**21.7%**	3.6%	1.4%	3.5%	Christian
Chad	2014	DHS	**44.4%**	**52.5%**	0.3%	0.0%	2.9%	Muslim
Côte d’Ivoire	2016	MICS	**41.0%**	**44.6%**	6.0%	0.7%	7.7%	Muslim
Ethiopia	2016	DHS	**67.5%**	**31.2%**	0.8%	0.6%	–	Christian
Ghana	2014	DHS	**80.2%**	**15.2%**	2.0%	0.0%	2.7%	Christian
Guinea-Bissau	2014	MICS	**31.5%**	**46.8%**	**17.7%**	0.6%	3.4%	Muslim
Liberia	2013	DHS	**86.2%**	**10.9%**	0.5%	0.0%	2.5%	Christian
Malawi	2015	DHS	**86.9%**	**12.5%**	–	0.1%	0.5%	Christian
Mozambique	2015	DHS	**60.4%**	**18.9%**	**12.2%**	2.0%	6.5%	Christian
Nigeria	2016	MICS	**40.4%**	**58.6%**	0.8%	0.1%	0.0%	Muslim
Sierra Leone	2013	DHS	**21.2%**	**78.5%**	0.0%	0.2%	0.1%	Muslim
Togo	2013	DHS	**60.7%**	**17.0%**	**14.3%**	0.1%	7.9%	Christian
Uganda	2016	DHS	**86.2%**	**12.9%**	0.0%	0.8%	0.1%	Christian

*Note:* in bold groups larger than 10%.

The Supplementary Material (Table S2 and Figures S1) show the distributions of wealth, urban/rural residence, and educational attainment by religious group in each country. In general, Christian women and their families were wealthier, more educated and more urban. For all countries but Uganda, higher proportions of women with at least secondary education were Christians. In Chad and Liberia, Muslim women were richer, and in Uganda, Muslims were richer, more educated, and more urban. In the four countries where Folk religion is common, this group was disadvantaged compared to the other two, except for Mozambique where the least advantaged group was Muslim.

**Fig. 1:**
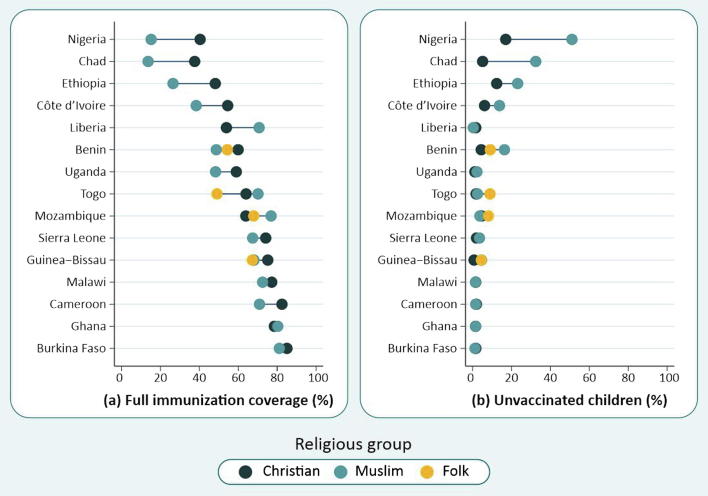
(a) Full immunization coverage and (b) unvaccinated children, by religious group. Countries are ranked by national full immunization coverage.

The average proportion of fully immunized children in all countries studied was 58.9%, ranging from 24.2% in Nigeria to 81.3% in Burkina Faso. Table 2 and Fig. 1a show that coverage among Christians was higher than for Muslims in 11 countries, in nine of which the differences were statistically significant. In four countries Muslims had higher coverage than Christians, of which two were significant (Liberia and Mozambique). Considering Folk religions, we found lower vaccination rates in Guinea-Bissau and Togo among this group in comparison to both Christian and Muslim categories, however, the rate was statistically significant only for Togo.

**Table 2:** Percentage of children fully immunized and unvaccinated at national level and by religious group.

Country	National			Christian			Muslim			Folk			Ratio Muslim/Christian	Ratio Folk/Christian	Ratio Folk/Muslim
	Percentage	95%CI	N	Percentage	95%CI	N	Percentage	95%CI	N	Percentage	95%CI	N			
**Full immunization**															
Benin	58.1%	(55.2%;61.0%)	2.439	62.9%	(59.3%;66.5%)	1.229	51.0%	(45.4%;56.5%)	735	55.4%	(48.2%;62.5%)	329	0.81 a	0.88	1.09
Burkina Faso	81.3%	(79.3%;83.4%)	2.791	85.0%	(81.8%;88.2%)	757	81.1%	(78.5%;83.7%)	1.741				0.95		
Cameroon	77.5%	(74.5%;80.6%)	1.389	82.5%	(79.4%;85.6%)	961	70.9%	(62.9%;79.0%)	318				0.86 a		
Chad	25.7%	(22.7%;28.6%)	2.880	37.6%	(32.5%;42.7%)	1.011	13.6%	(11.3%;16.0%)	1.770				0.36 a		
Côte d’Ivoire	44.9%	(41.1%;48.7%)	1.750	56.2%	(49.9%;62.4%)	540	40.3%	(35.9%;44.7%)	851				0.72 a		
Ethiopia	38.6%	(34.5%;42.8%)	1.929	48.2%	(43.0%;53.4%)	968	26.5%	(20.4%;32.5%)	921				0.55 a		
Ghana	78.2%	(74.6%;81.8%)	1.128	78.6%	(74.3%;82.8%)	806	80.3%	(73.5%;87.1%)	233				1.02		
Guinea-Bissau	69.6%	(65.6%;73.6%)	1.581	75.3%	(68.9%;81.7%)	322	68.2%	(62.9%;73.4%)	786	67.3%	(59.5%;75.0%)	390	0.91 a	0.89	0.99
Liberia	54.8%	(50.9%;58.8%)	1.433	53.9%	(49.8%;58.1%)	1.216	70.7%	(59.7%;81.8%)	163				1.31b		
Malawi	76.5%	(74.4%;78.6%)	3.248	77.2%	(74.9%;79.5%)	2.736	72.5%	(68.0%;77.1%)	488				0.94		
Mozambique	66.5%	(62.3%;70.8%)	1.029	63.9%	(58.3%;69.5%)	581	76.9%	(68.9%;84.9%)	182	67.9%	(60.0%;75.8%)	159	1.20b	1.06	0.88
Nigeria	24.2%	(22.3%;26.0%)	5.377	41.6%	(38.6%;44.7%)	2.152	15.9%	(13.8%;18.1%)	3.178				0.38 a		
Sierra Leone	68.4%	(65.2%;71.6%)	2.090	74.1%	(68.4%;79.8%)	387	67.4%	(63.9%;70.9%)	1.694				0.91 a		
Togo	61.5%	(57.5%;65.4%)	1.404	64.0%	(59.6%;68.4%)	688	70.2%	(64.2%;76.1%)	299	49.1%	(39.8%;58.3%)	293	1.10	0.77 a	0.70b
Uganda	57.3%	(55.0%;59.7%)	2.922	59.0%	(56.7%;61.3%)	2.492	48.3%	(41.8%;54.9%)	397				0.82 a		
**Unvaccinated children**															
Benin	8.8%	(7.0%;10.6%)	2.439	4.4%	(3.0%;5.9%)	1.229	16.4%	(11.4%;21.3%)	735	9.1%	(5.2%;12.9%)	329	3.69b	2.05c	0.55b
Burkina Faso	1.8%	(1.1%;2.5%)	2.791	1.7%	(0.4%;2.9%)	757	1.2%	(0.5%;1.9%)	1.741				0.75		
Cameroon	2.5%	(1.4%;3.5%)	1.389	2.0%	(0.8%;3.1%)	961	1.6%	(0.4%;2.8%)	318				0.81		
Chad	18.6%	(16.5%;20.7%)	2.880	5.2%	(3.6%;6.7%)	1.011	32.5%	(29.2%;35.8%)	1.770				6.29b		
Côte d’Ivoire	12.0%	(9.9%;14.2%)	1.750	6.1%	(3.8%;8.5%)	540	13.4%	(10.0%;16.7%)	851				2.18b		
Ethiopia	16.5%	(13.5%;19.4%)	1.929	12.4%	(8.9%;15.9%)	968	23.2%	(18.1%;28.2%)	921				1.87b		
Ghana	1.6%	(0.7%;2.5%)	1.128	1.5%	(0.5%;2.6%)	806	1.6%	(0.0%;3.8%)	233				1.03		
Guinea-Bissau	2.9%	(1.9%;3.9%)	1.581	0.4%	(0.0%;1.3%)	322	4.3%	(2.6%;6.1%)	786	2.4%	(0.7%;4.2%)	390	9.78b	5.54c	0.57
Liberia	1.5%	(0.8%;2.3%)	1.433	1.6%	(0.7%;2.6%)	1.216	0.4%	(0.0%;1.1%)	163				0.22		
Malawi	1.6%	(1.0%;2.2%)	3.248	1.6%	(1.0%;2.3%)	2.736	1.5%	(0.2%;2.8%)	488				0.93		
Mozambique	5.0%	(2.6%;7.4%)	1.029	4.7%	(1.9%;7.5%)	581	3.7%	(0.0%;7.7%)	182	8.1%	(2.1%;14.1%)	159	0.80	1.73	2.17
Nigeria	40.0%	(37.6%;42.3%)	5.416	17.0%	(14.8%;19.3%)	2.169	50.9%	(47.7%;54.2%)	3.196				3.00b		
Sierra Leone	3.5%	(2.3%;4.6%)	2.090	2.0%	(0.5%;3.6%)	387	3.5%	(2.4%;4.7%)	1.694				1.74		
Togo	3.6%	(2.3%;5.0%)	1.404	1.8%	(0.7%;2.9%)	688	2.5%	(0.7%;4.3%)	299	8.9%	(4.2%;13.7%)	293	1.41	5.04c	3.58c
Uganda	1.3%	(0.8%;1.8%)	2.922	1.2%	(0.7%;1.7%)	2.492	2.2%	(0.7%;3.8%)	397				1.92		

^a^Higher percentage among Christians (p value < 0.05 based on crude Poisson regression models).

^b^Higher percentage among Muslims (p < 0.05).

^c^Higher percentage among Folk practitioners (p < 0.05).

The average proportion of unvaccinated children in all countries studied was 8.1%, ranging from 1.3% in Uganda to 40.0% in Nigeria (Table 2 and Fig. 1b). In six of the 15 countries, there were significant differences according to religion. In all of these six countries, Muslim children were at higher risk of not receiving any vaccines, with ratios of unvaccinated children (Muslim/Christian) ranging from 1.87 in Ethiopia to 9.78 in Guinea-Bissau. In the six countries with the highest proportions of unvaccinated children, this was largely due to lack of immunizations among Muslims (Fig. 1b). Folk groups had significantly higher proportions of unvaccinated children than Christians in three countries, namely Benin, Guinea-Bissau, and Togo (Table 2 and Fig. 1b). In Benin, the Muslim group had a higher prevalence, compared to both Christians and Folk. In contrast, the estimates were significantly higher for Folk affiliates in Togo compared to the other two groups. No significant differences between Folk and Muslim groups were found in Guinea-Bissau. Mozambique, the fourth country with expressive proportion of Folk affiliates, did not presented any differences among the religious groups.

We also analyzed how coverage of the four separate vaccines varied according to religion (Supplementary Table S3). These patterns were very similar to those for full immunization coverage, suggesting that any differences between religious groups were not due to a particular vaccine. Coverage for BCG vaccine was not statistically associated with religion in three countries (Liberia, Mozambique, and Uganda) for which there was an association with full coverage, possibly because this vaccine is given soon after birth and does not require visiting a facility.

**Fig. 2:**
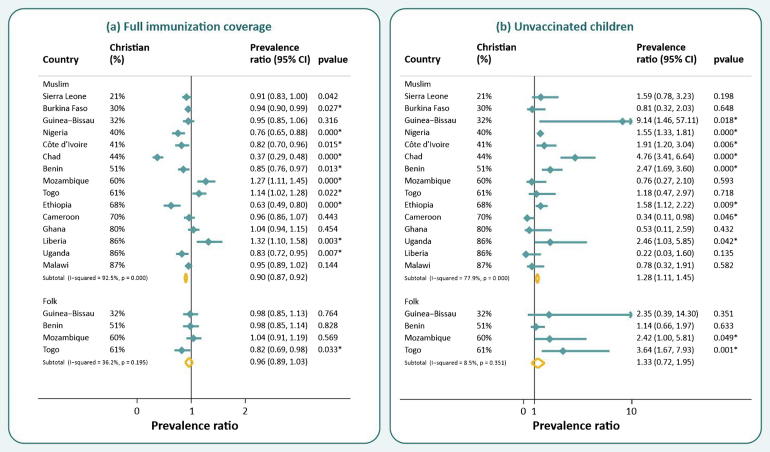
Coverage ratios adjusted for wealth, area of residence and education, and correspondent 95% confidence intervals for (a) full immunization coverage and (b) unvaccinated children from Muslim and Folk (when available) families compared to Christian, by country. Countries are ranked by proportion of Christian population.

The results of Poisson regression models are presented in Fig. 2 and in Supplementary Table S4. Full adjustment for women’s education, wealth, and urban/rural residence did not modify the results for most countries. Of the nine countries with significantly higher immunization coverage among Christians compared to Muslims in the crude analyses, seven remained significant. In Cameroon and Guinea-Bissau, the adjustment removed the effect of religion on vaccination coverage. Burkina Faso was the only country in which higher coverage among Christians became significant after adjustment. The difference was still significant for the two countries where Muslims had significantly higher unadjusted coverage. In Togo, Muslims had significantly higher coverages than both Christians and Folks. We performed a test of heterogeneity for the pooled prevalence ratios and found an I^2^ statistic of 92.5% (p value < 0.001) for the Muslim-Christian comparison and 14.8% (p = 0.318) for the Folk-Christian, suggesting greater heterogeneity across countries for the first paired comparison, but more homogeneity for the second.

Controlling for socio-demographic variables had some influence on the estimated associations between religious group and unvaccinated children. For the six countries with crude differences between Muslim and Christians, the association remained significant after full adjustment, but in four (Benin, Chad, Ethiopia, and Nigeria), there was substantial attenuation of the ratio, reducing the magnitude of the inequality. In Uganda, the difference became significant after adjustment, with a higher risk for Muslim children. Again, we found heterogeneity across countries for the Muslim-Christian comparison, but not for Folk-Christian ratios, with I^2^ statistics of 76.7% (p < 0.001) and 8.5% (p = 0.351), respectively.

In a set of ecological analyses, countries with higher proportions of Muslim women showed lower adjusted ratios for full vaccine coverage (Pearson’s r = -0.466, p = 0.080), indicating that the higher the Muslim population in a given country, lower the immunization coverage of Muslim children compared to Christians, and higher ratios for unvaccinated children (r = 0.358; p = 0.189). National immunization coverage was also directly associated with the adjusted ratios (r = 0.609; p = 0.016). In contrast, there was no such association between national coverage and the adjusted ratio for unvaccinated children (r = 0.079; p = 0.779).

Sex of the child was not found to consistently affect the likelihood of being vaccinated or not receiving any vaccine when the full national samples were studied (Table 3). In seven of the 15 countries, immunization coverage appeared to be higher among boys, but none of the differences were statistically significant. No country had significantly higher coverage for girls than for boys. For unvaccinated children, a significant sex difference was found only in Liberia where this was more common among boys than girls.

**Table 3:** Percentage of children fully immunized and unvaccinated, by sex.

Country	Boys			Girls			Sex ratio boys/girls	p value#
	Percentage	95%CI	N	Percentage	95%CI	N		
**Full immunization**								
Benin	59.6%	(55.9%;63.3%)	1.208	56.7%	(53.2%;60.2%)	1.231	1.05	0.201
Burkina Faso	82.1%	(79.5%;84.7%)	1.394	80.6%	(78.0%;83.3%)	1.397	1.02	0.388
Cameroon	76.1%	(72.3%;79.9%)	729	79.2%	(74.9%;83.5%)	660	0.96	0.241
Chad	25.4%	(22.0%;28.9%)	1.461	25.9%	(22.1%;29.7%)	1.419	0.98	0.823
Côte d’Ivoire	45.4%	(40.5%;50.3%)	896	44.4%	(39.8%;49.1%)	854	1.02	0.757
Ethiopia	36.5%	(31.0%;42.0%)	946	40.5%	(35.3%;45.7%)	983	0.90	0.250
Ghana	79.3%	(75.1%;83.5%)	577	77.1%	(72.5%;81.7%)	551	1.03	0.416
Guinea-Bissau	69.4%	(65.3%;73.6%)	816	69.7%	(63.8%;75.6%)	766	1.00	0.927
Liberia	52.9%	(47.9%;58.0%)	736	56.9%	(51.0%;62.8%)	697	0.93	0.320
Malawi	75.9%	(73.1%;78.7%)	1.622	77.1%	(74.4%;79.8%)	1.626	0.98	0.522
Mozambique	68.8%	(63.1%;74.5%)	491	64.3%	(58.4%;70.2%)	538	1.07	0.257
Nigeria	23.9%	(21.7%;26.2%)	2.653	24.4%	(21.7%;27.0%)	2.724	0.98	0.795
Sierra Leone	68.4%	(64.7%;72.1%)	1.016	68.4%	(64.0%;72.8%)	1.074	1.00	0.998
Togo	63.9%	(59.1%;68.7%)	720	59.0%	(54.2%;63.8%)	684	1.08	0.082
Uganda	58.4%	(55.6%;61.2%)	1.496	56.2%	(53.0%;59.5%)	1.426	1.04	0.278
**Unvaccinated children**								
Benin	8.4%	(6.1%;10.6%)	1.208	9.1%	(6.9%;11.4%)	1.231	0.92	0.578
Burkina Faso	1.6%	(0.8%;2.3%)	1.394	2.1%	(1.0%;3.1%)	1.397	0.74	0.338
Cameroon	2.6%	(1.0%;4.3%)	729	2.3%	(0.9%;3.6%)	660	1.16	0.739
Chad	17.4%	(14.9%;20.0%)	1.461	19.8%	(17.1%;22.5%)	1.419	0.88	0.143
Côte d’Ivoire	11.9%	(9.2%;14.6%)	896	12.2%	(9.2%;15.1%)	854	0.98	0.883
Ethiopia	16.8%	(13.2%;20.4%)	946	16.1%	(12%;20.2%)	983	1.04	0.790
Ghana	2.0%	(0.4%;3.6%)	577	1.2%	(0.4%;2.1%)	551	1.63	0.361
Guinea-Bissau	2.6%	(1.2%;4.0%)	816	3.2%	(1.8%;4.7%)	766	0.81	0.540
Liberia	2.2%	(0.8%;3.7%)	736	0.8%	(0.3%;1.2%)	697	2.87 a	0.016 *
Malawi	1.4%	(0.8%;2.1%)	1.622	1.7%	(0.9%;2.6%)	1.626	0.82	0.534
Mozambique	5.1%	(1.7%;8.5%)	491	4.9%	(2.6%;7.3%)	538	1.03	0.926
Nigeria	39.8%	(36.8%;42.7%)	2.668	40.2%	(37.1%;43.4%)	2.748	0.99	0.815
Sierra Leone	3.8%	(2.3%;5.3%)	1.016	3.2%	(1.9%;4.5%)	1.074	1.17	0.483
Togo	4.6%	(2.6%;6.6%)	720	2.7%	(1.1%;4.2%)	684	1.71	0.123
Uganda	1.1%	(0.6%;1.7%)	1.496	1.5%	(0.7%;2.3%)	1.426	0.75	0.450

^a^Percentage of boys significantly higher than girls.

# p value for Pearson's chi-squared test.

* p value < 0.05.

When examining sex differences within religious groups, a few significant differences were found (Table 4). Christian boys were less likely to be vaccinated than Christian girls in Cameroon. Regarding unvaccinated children, Christian girls were more likely not to be vaccinated than Christian boys in Burkina Faso, while in Liberia the proportion was higher among Christians boys. In total, eight significant differences were found in 68 independent comparisons by sex within religious group.

**Table 4:** Percentage of children full immunized and unvaccinated, by sex and religion.

Country	Religion	Boys			Girls			Sex ratio boys/girls	chi^2^ p value	Interaction p value#	
		Percentage	95%CI	N	Percentage	95%CI	N			Unadjusted	Adjusted
**Full immunization**											
Benin	Christian	63.8%	(59.0%;68.6%)	631	62.0%	(57.1%;66.8%)	598	1.03	0.559	..	..
	Muslim	52.8%	(45.6%;60.0%)	358	49.2%	(42.7%;55.8%)	377	1.07	0.385	0.674	0.908
	Folk	54.7%	(44.9%;64.5%)	145	55.9%	(47.1%;64.7%)	184	0.98	0.844	0.668	0.661
Burkina Faso	Christian	87.9%	(83.6%;92.2%)	366	82.3%	(78.0%;86.6%)	391	1.07	0.060	..	..
	Muslim	81.6%	(78.3%;85.0%)	876	80.6%	(77.1%;84.1%)	865	1.01	0.660	0.224	0.205
Cameroon	Christian	78.4%	(73.7%;83.0%)	497	87.1%	(83.4%;90.8%)	464	0.90b	0.003 *	..	..
	Muslim	74.9%	(66.3%;83.6%)	177	66.5%	(55.4%;77.6%)	141	1.13	0.152	0.020**	0.015**
Chad	Christian	37.8%	(32.1%;43.5%)	512	37.4%	(30.5%;44.3%)	499	1.01	0.920	..	..
	Muslim	12.5%	(9.6%;15.5%)	902	14.8%	(11.6%;17.9%)	868	0.85	0.261	0.339	0.517
Côte d’Ivoire	Christian	56.4%	(48.6%;64.2%)	273	55.9%	(48.2%;63.7%)	267	1.01	0.919	..	..
	Muslim	38.0%	(32.3%;43.8%)	438	42.6%	(36.4%;48.8%)	413	0.89	0.266	0.350	0.339
Ethiopia	Christian	46.2%	(39.1%;53.3%)	448	49.8%	(43.8%;55.8%)	520	0.93	0.364	..	..
	Muslim	25.1%	(17.0%;33.3%)	474	27.8%	(19.5%;36.0%)	447	0.91	0.639	0.913	0.953
Ghana	Christian	79.4%	(74.5%;84.2%)	413	77.7%	(72.4%;83.0%)	393	1.02	0.580	..	..
	Muslim	85.1%	(78.0%;92.2%)	120	75.5%	(64.6%;86.4%)	113	1.13	0.114	0.261	0.251
Guinea-Bissau	Christian	77.9%	(71.6%;84.2%)	174	72.0%	(60.8%;83.3%)	148	1.08	0.338	..	..
	Muslim	68.3%	(62.6%;73.9%)	411	68.1%	(60.6%;75.6%)	376	1.00	0.969	0.388	0.514
	Folk	64.2%	(53.8%;74.6%)	195	70.4%	(61.7%;79.2%)	195	0.91	0.277	0.154	0.285
Liberia	Christian	51.0%	(45.6%;56.4%)	624	57.0%	(50.6%;63.4%)	592	0.90	0.173	..	..
	Muslim	72.9%	(58.0%;87.9%)	84	68.1%	(52.0%;84.2%)	79	1.07	0.661	0.310	0.460
Malawi	Christian	76.6%	(73.6%;79.7%)	1.377	77.8%	(74.8%;80.9%)	1.359	0.98	0.556	..	..
	Muslim	71.2%	(64.2%;78.2%)	235	73.7%	(67.8%;79.6%)	253	0.97	0.595	0.793	0.863
Mozambique	Christian	67.8%	(60.1%;75.5%)	288	60.0%	(52.2%;67.7%)	293	1.13	0.146	..	..
	Muslim	75.9%	(64.4%;87.4%)	81	77.9%	(68.7%;87.0%)	101	0.97	0.769	0.244	0.379
	Folk	68.5%	(55.8%;81.2%)	70	67.4%	(55.9%;79.0%)	89	1.02	0.910	0.507	0.395
Nigeria	Christian	41.6%	(37.6%;45.6%)	1.063	41.7%	(37.8%;45.5%)	1.089	1.00	0.984	..	..
	Muslim	15.5%	(12.8%;18.2%)	1.561	16.3%	(13.0%;19.6%)	1.617	0.95	0.697	0.715	0.928
Sierra Leone	Christian	73.0%	(65.3%;80.6%)	189	75.2%	(67.3%;83.2%)	198	0.97	0.673	..	..
	Muslim	67.5%	(63.4%;71.7%)	824	67.3%	(62.6%;72.0%)	870	1.00	0.943	0.688	0.616
Togo	Christian	66.8%	(60.8%;72.8%)	341	61.4%	(55.6%;67.3%)	347	1.09	0.179	..	..
	Muslim	72.0%	(64.6%;79.4%)	168	67.7%	(58.0%;77.3%)	131	1.06	0.477	0.840	0.966
	Folk	48.5%	(38.1%;59.0%)	156	49.6%	(38.6%;60.7%)	137	0.98	0.844	0.407	0.563
Uganda	Christian	60.1%	(57.2%;62.9%)	1.264	57.9%	(54.5%;61.3%)	1.228	1.04	0.300	..	..
	Muslim	49.7%	(41.1%;58.3%)	214	46.7%	(37.7%;55.7%)	183	1.06	0.613	0.842	0.828
**Unvaccinated children**											
Benin	Christian	3.7%	(2.0%;5.5%)	631	5.1%	(3.0%;7.3%)	598	0.73	0.281	..	..
	Muslim	17.0%	(10.8%;23.3%)	358	15.8%	(10.1%;21.4%)	377	1.08	0.713	0.261	0.140
	Folk	8.4%	(3.6%;13.2%)	145	9.6%	(3.8%;15.4%)	184	0.88	0.760	0.741	0.709
Burkina Faso	Christian	0.0%	(0.0%;0.0%)	366	3.3%	(0.8%;5.7%)	391	0.00b	0.012 *	..	..
	Muslim	1.4%	(0.5%;2.2%)	876	1.1%	(0.0%;2.3%)	865	1.20	0.765	0.000**	–
Cameroon	Christian	2.0%	(0.3%;3.7%)	497	2.0%	(0.4%;3.6%)	464	0.99	0.990	..	..
	Muslim	1.4%	(0.1%;2.7%)	177	1.8%	(0.0%;3.8%)	141	0.79	0.748	0.809	0.794
Chad	Christian	4.6%	(2.9%;6.2%)	512	5.8%	(3.4%;8.2%)	499	0.79	0.368	..	..
	Muslim	31.1%	(26.8%;35.4%)	902	33.9%	(29.7%;38.1%)	868	0.92	0.296	0.592	0.735
Côte d’Ivoire	Christian	8.2%	(4.3%;12.0%)	273	4.1%	(1.7%;6.5%)	267	2.01	0.067	..	..
	Muslim	12.5%	(8.3%;16.6%)	438	14.3%	(9.7%;18.9%)	413	0.87	0.531	0.062**	0.065**
Ethiopia	Christian	11.5%	(7.1%;15.8%)	448	13.1%	(8.8%;17.5%)	520	0.87	0.525	..	..
	Muslim	24.4%	(18.4%;30.3%)	474	22.0%	(14.1%;30.0%)	447	1.11	0.643	0.439	0.446
Ghana	Christian	1.6%	(0.0%;3.4%)	413	1.5%	(0.4%;2.6%)	393	1.02	0.978	..	..
	Muslim	2.8%	(0.0%;7.1%)	120	0.4%	(0.0%;1.1%)	113	7.31	0.073	0.177	0.154
Guinea-Bissau	Christian	0.8%	(0.0%;2.4%)	174	0.0%	(0.0%;0.0%)	148	0.00	0.371	..	..
	Muslim	3.9%	(1.5%;6.3%)	411	4.8%	(2.4%;7.2%)	376	0.82	0.605	–	0.000**
	Folk	1.6%	(0.0%;3.3%)	195	3.3%	(0.8%;5.8%)	195	0.49	0.208	–	0.000**
Liberia	Christian	2.7%	(0.9%;4.4%)	624	0.6%	(0.1%;1.0%)	592	4.71 a	0.002 *	..	..
	Muslim	0.0%	(0.0%;0.0%)	84	0.8%	(0.0%;2.3%)	79	0.00	0.286	0.000**	0.000**
Malawi	Christian	1.2%	(0.6%;1.9%)	1.377	2.0%	(0.9%;3.0%)	1.359	0.62	0.179	..	..
	Muslim	2.5%	(0.2%;4.9%)	235	0.6%	(0.0%;1.8%)	253	4.14	0.170	0.109	0.110
Mozambique	Christian	3.8%	(0.3%;7.4%)	288	5.6%	(2.2%;8.9%)	293	0.69	0.428	..	..
	Muslim	6.1%	(0.0%;13.8%)	81	1.4%	(0.0%;3.4%)	101	4.32	0.101	0.069**	0.112
	Folk	8.0%	(1.0%;14.9%)	70	8.3%	(0.6%;15.9%)	89	0.97	0.947	0.629	0.485
Nigeria	Christian	17.9%	(15.0%;20.9%)	1.063	16.1%	(13.1%;19.1%)	1.098	1.12	0.357	..	..
	Muslim	50.4%	(46.3%;54.5%)	1.561	51.5%	(47.1%;55.8%)	1.630	0.98	0.692	0.301	0.344
Sierra Leone	Christian	2.1%	(0.0%;4.5%)	189	2.0%	(0.0%;3.9%)	198	1.07	0.933	..	..
	Muslim	3.9%	(2.2%;5.5%)	824	3.2%	(1.8%;4.6%)	870	1.21	0.513	0.887	0.991
Togo	Christian	2.7%	(0.9%;4.4%)	341	0.9%	(0.0%;2.1%)	347	2.80	0.096	..	..
	Muslim	2.0%	(0.0%;4.1%)	168	3.1%	(0.1%;6.1%)	131	0.65	0.545	0.129	0.091**
	Folk	11.1%	(4.1%;18%)	156	6.7%	(0.3%;13.0%)	137	1.66	0.367	0.539	0.447
Uganda	Christian	0.9%	(0.3%;1.4%)	1.264	1.5%	(0.6%;2.3%)	1.228	0.60	0.239	..	..
	Muslim	2.5%	(0.4%;4.7%)	214	1.9%	(0.0%;4.3%)	183	1.34	0.711	0.374	0.324

^a^Percentage among boys significantly higher than among girls.

^b^Percentage among girls significantly higher than among boys.

* p value < 0.05 for chi-squared test.

** p value < 0.10 for interaction coefficient of Poisson regression model.

# p value based on interaction test using Poisson regression models; references: Male and Christian categories.

Formal tests of interaction between child sex and religion were carried out for all crude and adjusted comparisons. With a p-level of 0.10 we found four statistically significant results out of 39 independent comparisons (Table 4 and Supplementary Table S5).

The widest religious-group disparities were found in countries with low overall immunization coverage, namely Chad, Ethiopia and Nigeria, suggesting that the Muslim disadvantage was more evident in low-coverage countries (figures and coefficients of correlation are presented in the Supplementary Figure S3 and Table S6). Also, countries with higher proportions of Muslim population tended to show lower Muslim-Christian vaccination coverage ratios (Supplementary Table S6).

## 4 Discussion

The global health literature is remarkably scant on the role of religion in explaining social inequalities in health. Even SDG 17.18, which calls for disaggregated analyses of national performance, does not include religion as one of the suggested variables for monitoring inequalities. Yet, religion may affect health outcomes through direct influences on beliefs and values, or due to differential access to resources [8], [19]. Religion may also contribute to improved mental health and health-related behaviors [20]. It is noteworthy, however, that epidemiological studies often treat religion solely as a confounding variable, without detailed examination of potential effects and their pathways [19], [21]. In light of the importance of religion for health, it is rather surprising that data from national surveys have not been subjected to multi-country analyses of health inequalities. We have contributed to the lack of systematic, multi-country studies on religion and health-related behaviors by analyzing immunization coverage among African children from 15 countries in SSA where both Muslims and Christians comprised 10% or more of the survey sample.

Eighty four percent of the world’s population is estimated to be affiliated to a religion, and Christian and Muslim faiths are the most common [11]. Sub-Saharan Africa is home to about 24% of all the Christians and 15% of all Muslims in the world [11]. The 15 countries included in the analyses account for 50% of the population in Sub-Saharan Africa, and have median values of 53% for Christian, 24% for Muslim and 3% for Folk affiliation, compared to 63%, 30% and 3% for the whole continent. Among the 19 countries with a survey that were not included, one (Guinea) did not provide information on vaccines, and the remaining had fewer than 10% of the sample in one of the two main groups (Christian and Muslim) [11].

Religious diversity leads to differences in life conditions. In our analyses, Muslim and Folk women were less urbanized, had lower formal education and were more likely to live in poverty than Christian women.

Overall, the average proportion of fully vaccinated children in the 15 countries was only 58%, and unvaccinated children accounted for 8%. Yet, national averages often mask inequalities, and disaggregated analyses are essential. Most countries presented higher immunization coverage among children of Christian families compared to Muslim families, and a few showed higher coverage among Muslims. The widest religious-group disparities were found in countries with low overall immunization coverage, namely Chad, Ethiopia and Nigeria, suggesting that when coverage is high, both Christian and Muslim children are reached.

We tested the hypothesis that countries where Muslims constituted most of the population would show more favorable results for children from Muslim families than would be the case for countries where Muslims are a minority. Yet, our results were in the opposite direction (Supplementary Table S6), although the correlation was not significant (p value = 0.08 in the adjusted analyses). Further studies are needed to understand this apparent contradiction.

Our results on unvaccinated children were quite striking. Although significant differences were observed for fewer countries, Muslim children represented a substantial proportion of those who were not vaccinated in Chad, Ethiopia and Nigeria. Religious-group gaps were attenuated in several countries after adjustment for wealth, women’s education and residence, but associations remained significant in most countries, and in a few cases (Burkina Faso and Togo for full immunization and Uganda for unvaccinated children) differences became significant only after adjustment.

The largest difference between crude and adjusted coefficients for immunization coverage was observed in Nigeria, where the Muslim-Christian prevalence ratio changed from 0.38 to 0.73, suggesting that much of the religious disparity in immunization in this country is associated with socioeconomic factors. The same patterns were observed for unvaccinated children in this country. Earlier studies found that 80% of non-immunized children in Nigeria were born to Muslim mothers, who are concentrated in the North of the country, the poorest region [22] known for being historically resistant to vaccination and other “Western” health interventions [23], [24]. In early 2000s, polio vaccine was held by Muslim leaders as being used to sterilize young girls, which led to vaccine hesitancy and low coverage, with a resulting polio epidemic that spread to polio-free countries in Africa, Asia and the Middle- East [25].

Our analyses showed similar patterns for all vaccines, except possibly for BCG which is given soon after birth and may be less prone to parental hesitancy than vaccines given at later ages, which require contact with a health facility or worker.

Both unadjusted and adjusted analyses are of value. The former is useful for targeting interventions at specific population subgroups (in this case, Muslim children) who are being left behind, whatever the reasons for these disparities. Adjusted analyses are helpful to answer the question of the extent by which poverty, lack of parental education, and place of residence may account for gaps in coverage.

Lower immunization rates among certain religious groups has been found to be associated with factors such as limited access to social programs, marginalization, limited knowledge and perceptions of illness, and influence of religious leaders [26], [27]. Further studies are needed to elucidate these mechanisms.

In addition to Nigeria, our literature review found that Muslim religion has been identified as a potential barrier to immunization coverage in Ethiopia [29] and in Chad [30], which along with Nigeria are the countries we identified with high proportions of unvaccinated Muslim children. Nevertheless, it has been argued that skepticism towards immunization by religious leaders are rooted less in faith-based beliefs than in health-related concerns that spread across social networks within religious communities [28], [31]. Islamic groups in other countries also have concerns that some vaccines are not *halal*, and therefore could not be administered [32]. On the other hand, a multi-country study on vaccine confidence showed that Muslim faith itself is not always linked to low coverage, citing Saudi Arabia as an example, where 100% of the respondents were Muslim and there were few objections to vaccines [28]. It is worth noting, however, that Saudi Arabia is a very wealthy country where economic conditions may offset any potential resistance to vaccination [33]. In fact, involvement of Islamic and Christian leaders in the promotion of child survival interventions in Sierra Leone was linked to a marked increase in coverage in the 1980s [31]. Similar successful experiences also took place in Angola and India [31].

Our analyses of gender bias within the different religious groups, and of interactions between sex of the child and religious groups, failed to show any clear patterns. Thus, our analyses did not provide evidence of preferential treatment of boys, either overall or within each religious group. However, even in the absence of sex differences in the two outcomes, gender issues remain as important determinants of access to preventive and curative interventions for children. Gender equality, expressed in women’s empowerment and decision-making power, is associated with better health outcomes for both their male and their female children [34], [35]. Again, national averages may mask variations at geographic and other sociodemographic sublevels even within religious groups; thus, the results might be interpreted taking this limitation into account [36].

The strengths of the present analyses include the comparability of DHS and MICS in terms of nationally-representative sampling and health indicator definitions [13]. With 15 countries where the comparison between Christians and Muslims was possible, our analyses provide the largest set of results on within-country religious inequalities in child health.

Several limitations must be acknowledged. Using surveys to assess the impact of religious affiliation on health is challenging. Although both MICS and DHS collect information on religion, different questions are used, and we had to assume that religion of the woman being interviewed (DHS) and that of the head of the household (MICS) was the same. Also, as expected, the numbers and labels of religious affiliations vary markedly from country to country. Differences in the geographic distribution of religious groups within a country were not studied, and access to vaccines and providers vary according to geography [37]. We opted not to adjust P levels for multiple comparisons given that separate samples were studied in each country, and that appropriate meta-analytic techniques were used to pool results.

Heterogeneity within the main religious denominations – for example between mainstream Christians and evangelical churches – was not considered in our analyses, yet major differences may occur. However, given our objective of presenting a multi-country comparison, we needed to rely on broad, consistent denominations; our results may be interpreted as a weighted average of the different affiliations in each broad category [8]. Further disaggregation into various categories would reduce the number of observations in each, and except for very large surveys this would not be advisable due to loss of analytic power. Lastly, available data do not allow differentiation between affiliation and individual beliefs and practices, as the questionnaires do not include information on religious attendance or subjective views of the religion. In SSA, individuals who report Christian and Islamic affiliation may also incorporate elements of African traditional religions into their daily lives [9].

In spite of the limitations, this multi-country study provided new insights into understanding the role of religion in child health, answering the SDG 17.18 demand for disaggregated data analyses. Taking together the results from the 15 countries, we found lower immunization coverage among Muslims than Christians, a finding that persisted after adjustment for wealth, education and residence in nearly all countries. Only four countries had a substantial proportion of folk practitioners, and there were no consistent patterns for this group compared to the other two.

Religion cannot be understood as separate from the cultural, economic, and political spheres of life that determine gender bias and access to life-saving interventions. Nevertheless, our analyses suggest that religious-group disparities are only partly due to measurable sociodemographic differences, suggesting that discrimination or parental hesitancy, possibly magnified through social networks, may play an important role. Greater involvement of Muslim leaders in vaccine promotion has proven to be effective in earlier studies and may constitute an important strategy for reaching children who are not vaccinated in selected countries.

Further qualitative and quantitative research is needed to explore the inequalities across religious groups that were identified in the present analyses, by using more granular definitions of religious affiliation and collecting information on practices and beliefs. Incorporation of religion in stratified analyses of child health indicators is essential for supporting the *leave no one behind* agenda of the Sustainable Development Goals.

## Ethics approval

Data used are from public sources of information and ethical approval has already been obtained by the institutions responsible in each country.

## Author’s contributions

JCC and CGV conceptualized the paper. JCC conducted the analyses, interpreted the results, and wrote the manuscript. CGV, GLD, SA, and AMW supervised the analysis, interpretation, and writing. All authors read and approved the final manuscript.

## Funding

Bill and Melinda Gates Foundation (Grant Number: OPP1148933), Wellcome Trust, and Associação Brasileira de Saúde Coletiva. The funders had no role in the data analysis, data interpretation, or writing of the paper. The corresponding author had full access to all the data and had final responsibility for the decision to submit for publication.

## Declaration of Competing Interest

The authors declare that they have no known competing financial interests or personal relationships that could have appeared to influence the work reported in this paper.
