# Supplementary material for: Religious affiliation and immunization coverage in 15 countries in Sub-Saharan Africa
Source: Vaccine. 2020 Jan 29;38(5):1160–9. doi: 10.1016/j.vaccine.2019.11.024 (PMC6995994; doi:10.1016/j.vaccine.2019.11.024)
Supplement: Supplementary data 1 [file mmc1.docx]

**Supplementary Material**

**Supplementary table S1 – Categories of the original variables recoded into eight religious groups, Demographic and Health Surveys and Multiple Indicator Cluster Surveys.**

| **Country (Year)**  **Source** | **Christian** | **Muslim** | **Folk** | **Other** | **Unaffiliated** |
| --- | --- | --- | --- | --- | --- |
| **Benin (2014)**  **MICS** | Catholique  Protestant méthodist  Autres protestants  Céleste  Autres chrétiens | Islam | Vodoun  Autres traditionnelles | Autre religion | Pas de religion |
| **Burkina Faso (2010)**  **DHS** | Catholic  Protestant | Muslim | Traditional/animist | Other | No religion |
| **Cameroon (2014)**  **MICS** | Catholique  Protestant  Autre chétien | Musulman | Animiste | Autre religion (non chrétien) | Pas de religion |
| **Chad (2014)**  **DHS** | Catholic  Protestant  Other Christians | Muslim | Animist | Other | No religion |
| **Côte d’Ivoire (2016)**  **MICS** | Catholique  Evangélique  Méthodiste  Céleste  Harriste  Autre religion chrétienne | Musulman | Animiste | Autre religion | Pas de religion |
| **Ethiopia (2016)**  **DHS** | Orthodox  Catholic  Protestant | Muslin | Traditional | Other | NA |
| **Ghana (2014)**  **DHS** | Catholic  Anglican  Methodist  Presbyterian  Pentecostal/Charismatic  Other Christian | Islam | Traditional/spiritualist | Other | No religion |
| **Guinea-Bissau (2014)**  **MICS** | Católica  Evangélico | Muçulmano | Anemista | Outra religião | Sem religião |
| **Liberia (2013)**  **DHS** | Christian | Muslim | Traditional religion | Other | No religion |
| **Malawi (2015)**  **DHS** | Christian  CCAP  Anglican  Seventh Day Adventist / Baptist  Other Christian | Muslim | NA | Other | No religion |
| **Mozambique (2015)**  **DHS** | Catholic  Protestant  Evangelical/ Pentecostal  Anglican | Islamic | Zion | Other | No religion |
| **Nigeria (2016)**  **MICS** | Christianity | Islam | Traditional | Other religion | No religion |
| **Sierra Leone (2013)**  **DHS** | Christian | Islam | Traditional | Bahai | None |
| **Togo (2013)**  **DHS** | Catholic  Evangelical Presbyterian  Methodist  Assembly of God  Baptist  Pentecotist  Jeovah Witness  Adventist  Other Christian | Muslim | Traditional/ animist | Other | No religion |
| **Uganda (2016)**  **DHS** | Anglican  Catholic  Seventh Day Adventist  Orthodox  Pentecostal/Born Again/ Evangelical  Baptist  Presbyterian  Mammon  Jeovah’s Witness  Salvation Army | Muslim | Traditional | Other | No religion |

**Supplementary table S2 – Distribution of education, wealth and residence according to religious groups, by country.**

| **Country** | **Religion** | **Women's education** | | | | **Wealth quintiles** | | | | | | **Area of residence** | | |
| --- | --- | --- | --- | --- | --- | --- | --- | --- | --- | --- | --- | --- | --- | --- |
|  |  | **None** | **Primary** | **Secondary+** | **p value^#^** | **Poorest** | **Poorer** | **Middle** | **Richer** | **Richest** | **p value^#^** | **Urban** | **Rural** | **p value^#^** |
| Benin | Muslim | 68.8% | 16.9% | 14.3% | <0.001* | 21.9% | 17.3% | 19.7% | 19.7% | 21.4% | <0.001* | 46.4% | 53.7% | <0.001* |
|  | Christian | 48.1% | 29.3% | 22.6% |  | 10.8% | 14.1% | 18.6% | 24.7% | 31.8% |  | 56.7% | 43.4% |  |
|  | Folk | 63.3% | 23.9% | 12.8% |  | 28.1% | 27.6% | 25.2% | 14.2% | 4.9% |  | 31.4% | 68.6% |  |
| Burkina Faso | Muslim | 78.9% | 11.6% | 9.4% | <0.001* | 15.3% | 18.0% | 20.4% | 21.8% | 24.5% | <0.001* | 26.0% | 74.0% | <0.001* |
|  | Christian | 58.6% | 19.7% | 21.7% |  | 15.8% | 18.2% | 16.1% | 18.1% | 32.0% |  | 35.4% | 64.6% |  |
| Cameroon | Muslim | 48.4% | 31.4% | 20.3% | <0.001* | 21.1% | 21.5% | 22.5% | 20.7% | 14.2% | <0.001* | 47.7% | 52.3% | 0.066 |
|  | Christian | 9.7% | 31.0% | 59.3% |  | 12.5% | 15.8% | 19.2% | 23.1% | 29.4% |  | 57.4% | 42.6% |  |
| Chad | Muslim | 83.7% | 10.0% | 6.4% | <0.001* | 14.8% | 17.2% | 21.2% | 23.3% | 23.5% | <0.001* | 25.8% | 74.2% | 0.392 |
|  | Christian | 36.6% | 37.9% | 25.5% |  | 23.4% | 21.7% | 17.9% | 16.3% | 20.7% |  | 22.6% | 77.4% |  |
| Côte d'Ivoire | Muslim | 20.0% | 55.9% | 24.1% | 0.011* | 13.7% | 17.5% | 22.3% | 25.5% | 21.0% | <0.001* | 58.4% | 41.6% | 0.757 |
|  | Christian | 20.4% | 52.3% | 27.3% |  | 13.3% | 14.7% | 14.6% | 21.7% | 35.7% |  | 59.5% | 40.5% |  |
| Ethiopia | Muslim | 57.0% | 35.3% | 7.7% | <0.001* | 23.0% | 22.7% | 20.9% | 17.0% | 16.4% | <0.001* | 13.8% | 86.2% | <0.001* |
|  | Christian | 42.9% | 35.3% | 21.8% |  | 13.0% | 15.8% | 18.3% | 21.3% | 31.6% |  | 26.4% | 73.6% |  |
| Ghana | Muslim | 41.5% | 18.3% | 40.2% | <0.001* | 27.0% | 19.6% | 17.1% | 21.1% | 15.3% | <0.001* | 55.6% | 44.5% | 0.907 |
|  | Christian | 13.0% | 17.1% | 69.9% |  | 12.1% | 16.7% | 21.6% | 23.6% | 26.0% |  | 54.9% | 45.1% |  |
| Guinea-Bissau | Muslim | 54.6% | 28.6% | 16.8% | <0.001* | 6.8% | 22.5% | 27.1% | 22.5% | 21.2% | <0.001* | 45.6% | 54.4% | <0.001* |
|  | Christian | 17.4% | 29.8% | 52.8% |  | 11.5% | 10.3% | 10.1% | 28.2% | 39.9% |  | 74.2% | 25.8% |  |
|  | Folk | 47.2% | 38.1% | 14.8% |  | 53.1% | 19.5% | 12.6% | 8.4% | 6.4% |  | 21.6% | 78.4% |  |
| Liberia | Muslim | 52.8% | 25.4% | 21.8% | <0.001* | 14.5% | 19.0% | 16.8% | 19.4% | 30.4% | 0.379 | 53.8% | 46.2% | 0.219 |
|  | Christian | 30.3% | 31.5% | 38.3% |  | 16.8% | 17.2% | 19.2% | 23.1% | 23.8% |  | 62.3% | 37.7% |  |
| Malawi | Muslim | 23.7% | 62.5% | 13.8% | <0.001* | 24.6% | 21.6% | 19.6% | 17.9% | 16.3% | <0.001* | 13.0% | 87.0% | 0.005* |
|  | Christian | 10.2% | 62.1% | 27.6% |  | 18.4% | 18.7% | 18.8% | 19.3% | 24.8% |  | 19.1% | 80.9% |  |
| Mozambique | Muslim | 32.0% | 52.0% | 16.0% | <0.001* | 25.7% | 23.1% | 17.9% | 19.4% | 13.9% | <0.001* | 40.3% | 59.7% | 0.134 |
|  | Christian | 24.4% | 50.0% | 25.6% |  | 19.4% | 17.6% | 16.6% | 18.9% | 27.6% |  | 37.1% | 62.9% |  |
|  | Folk | 29.1% | 58.4% | 12.6% |  | 10.5% | 15.7% | 24.8% | 29.5% | 19.6% |  | 26.0% | 74.0% |  |
| Nigeria | Muslim | 46.8% | 19.0% | 34.3% | <0.001* | 24.8% | 22.8% | 20.3% | 17.3% | 14.8% | <0.001* | 32.9% | 67.1% | 0.001* |
|  | Christian | 11.4% | 20.3% | 68.2% |  | 7.1% | 13.2% | 18.4% | 25.3% | 36.1% |  | 40.9% | 59.1% |  |
| Sierra Leone | Muslim | 60.4% | 14.4% | 25.2% | <0.001* | 19.6% | 19.0% | 20.3% | 21.1% | 20.1% | <0.001* | 31.9% | 68.1% | <0.001* |
|  | Christian | 38.5% | 12.5% | 49.0% |  | 15.0% | 15.7% | 13.1% | 17.7% | 38.5% |  | 49.5% | 50.5% |  |
| Togo | Muslim | 39.7% | 28.9% | 31.4% | <0.001* | 14.2% | 13.2% | 15.6% | 27.4% | 29.7% | <0.001* | 54.4% | 45.6% | <0.001* |
|  | Christian | 18.7% | 36.4% | 44.8% |  | 9.4% | 13.6% | 18.1% | 26.5% | 32.5% |  | 54.8% | 45.2% |  |
|  | Folk | 66.7% | 26.3% | 7.1% |  | 43.9% | 31.4% | 18.8% | 4.7% | 1.3% |  | 6.9% | 93.1% |  |
| Uganda | Muslim | 7.5% | 50.1% | 42.4% | <0.001* | 10.1% | 15.0% | 14.2% | 22.0% | 38.8% | <0.001* | 36.0% | 64.0% | <0.001* |
|  | Christian | 9.8% | 58.5% | 31.7% |  | 18.7% | 18.8% | 19.2% | 19.7% | 23.7% |  | 25.5% | 74.5% |  |

^#^ p values based on Pearson’s chi-squared test, * p value < 0.05

**Supplementary figures S1 – Distribution of educational attainment, wealth quintiles and urban/rural residence according to religious group, by country.**

| **Benin**  **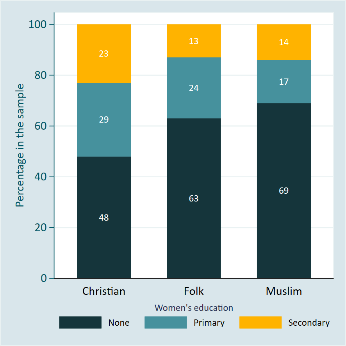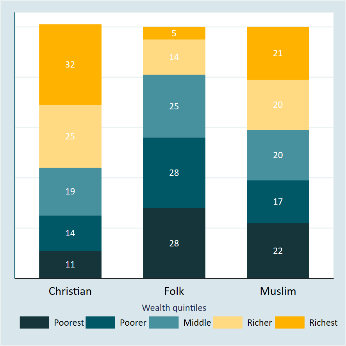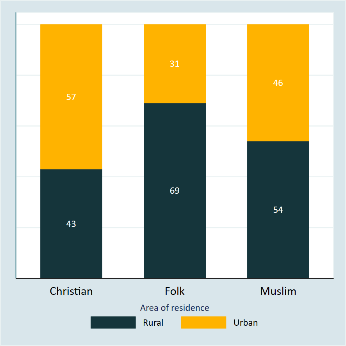** | **Burkina Faso**  **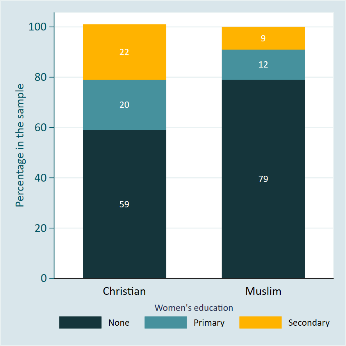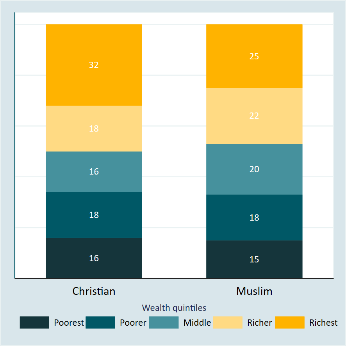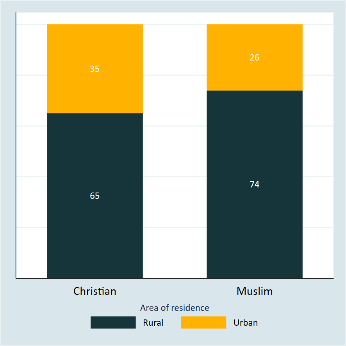** |
| --- | --- |
| **Cameroon**  **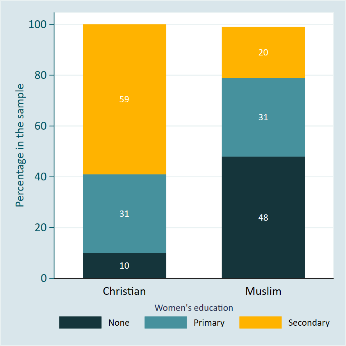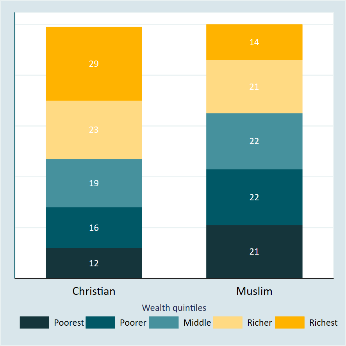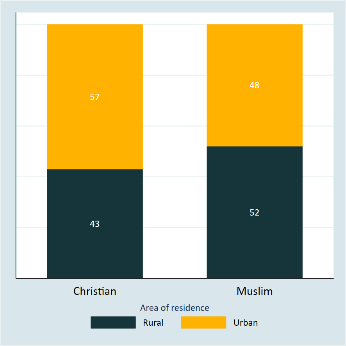** | **Chad**  **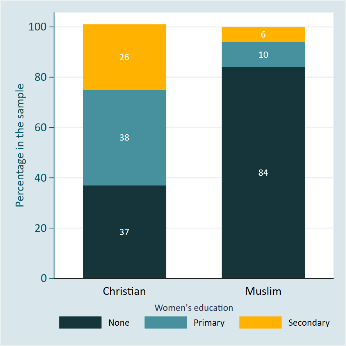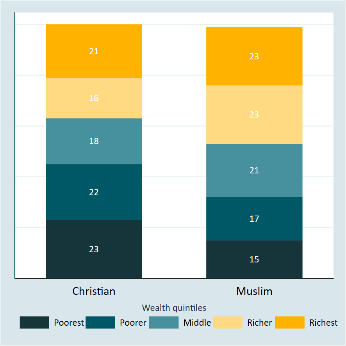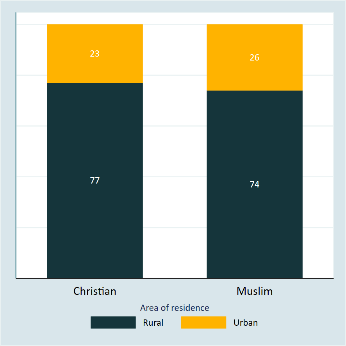** |
| **Côte d’Ivoire**  **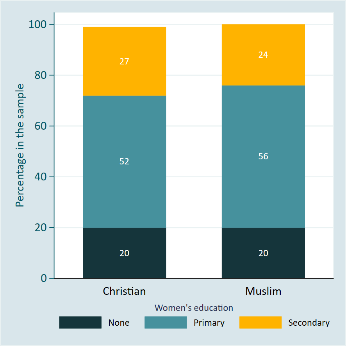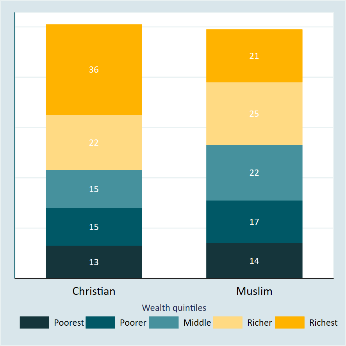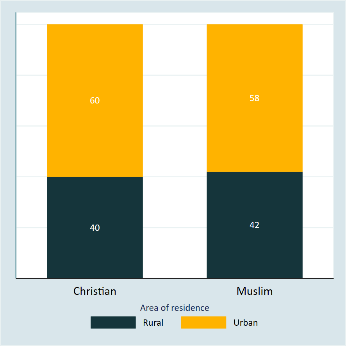** | **Ethiopia**  **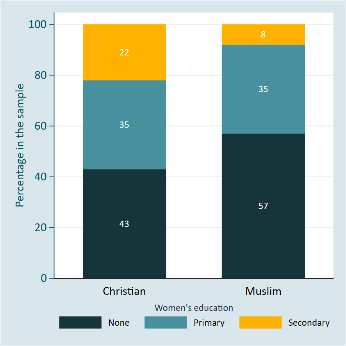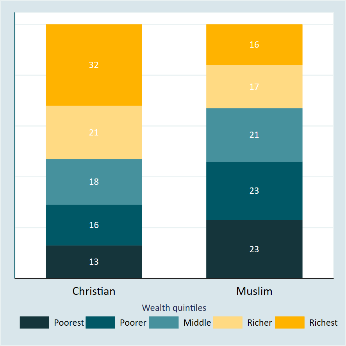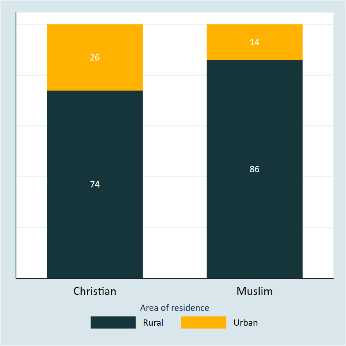** |
| **Ghana**  **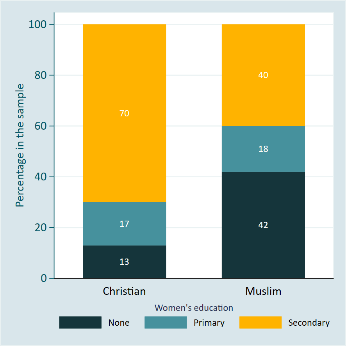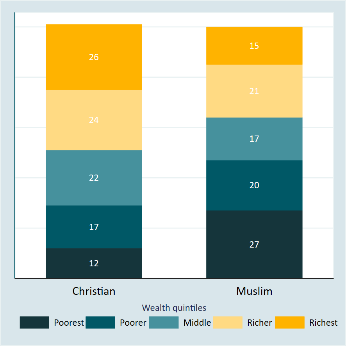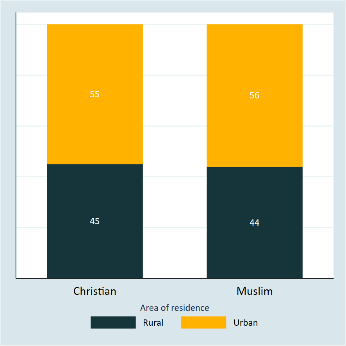** | **Guinea-Bissau**  **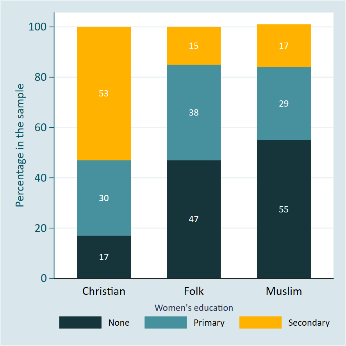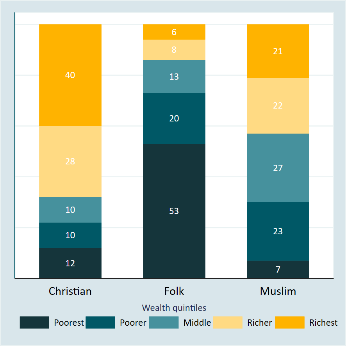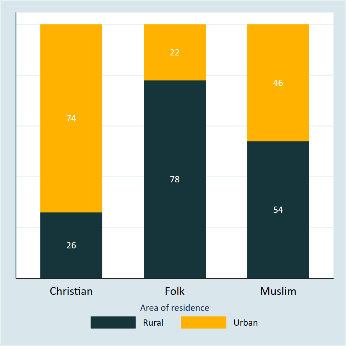** |
| **Liberia**  **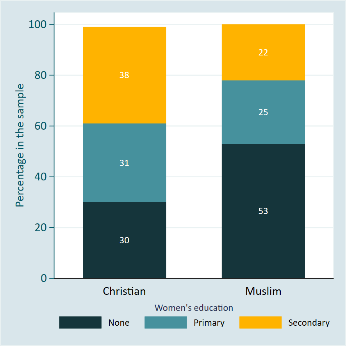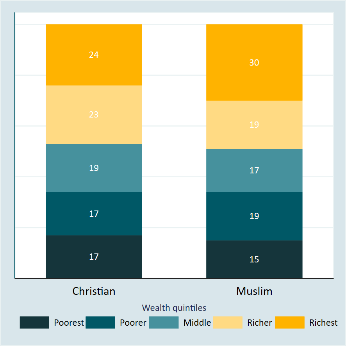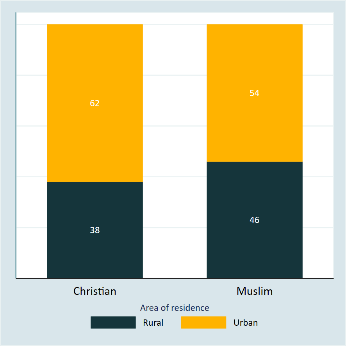** | **Malawi**  **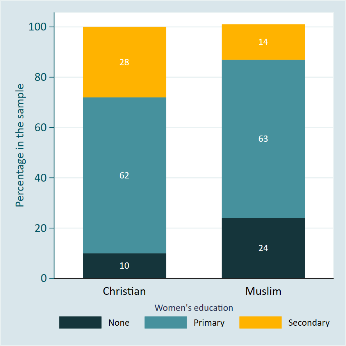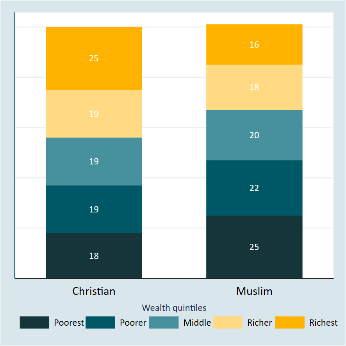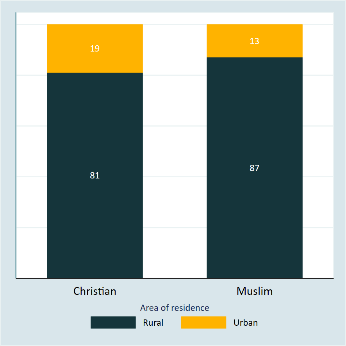** |
| **Mozambique**  **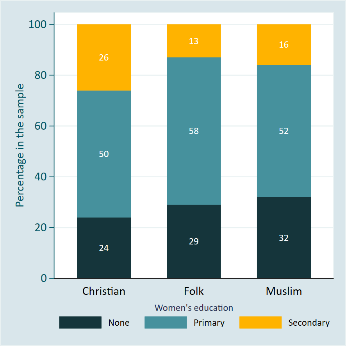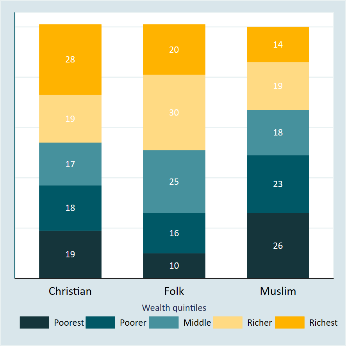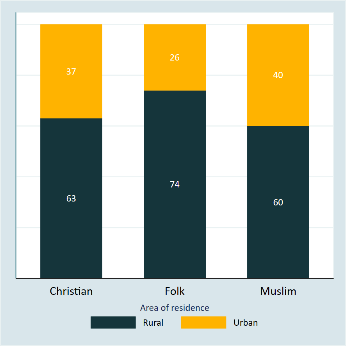** | **Nigeria**  **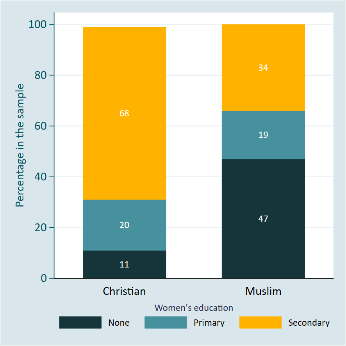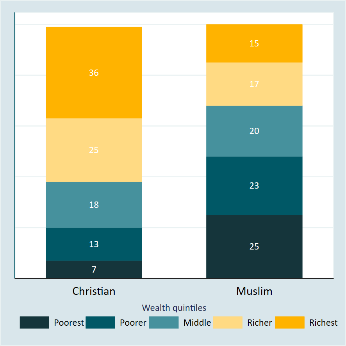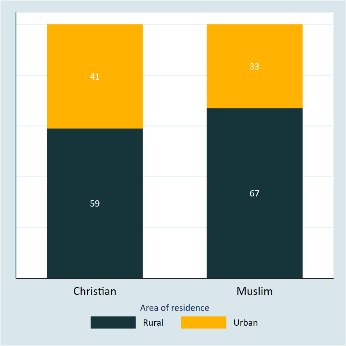** |
| **Sierra Leone**  **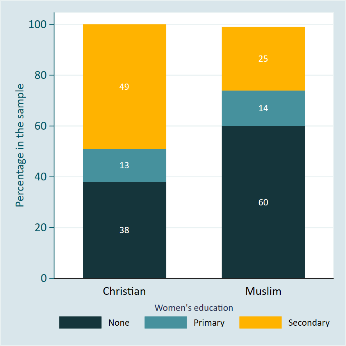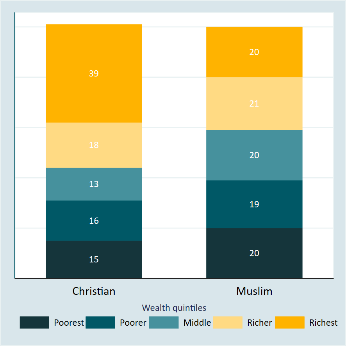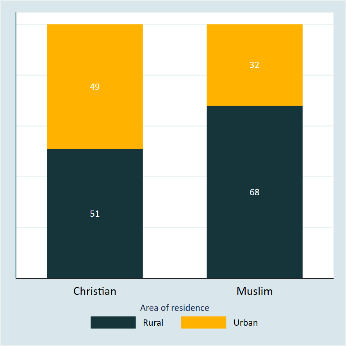** | **Togo**  **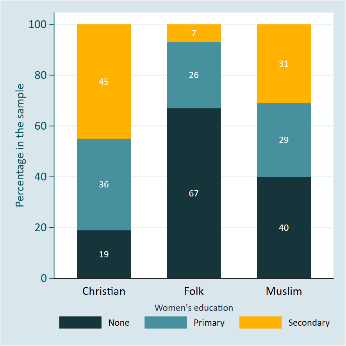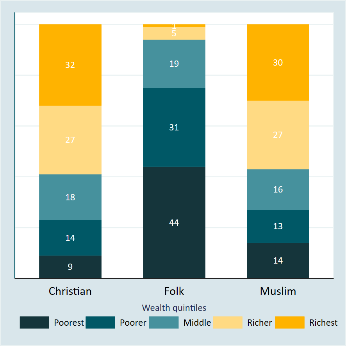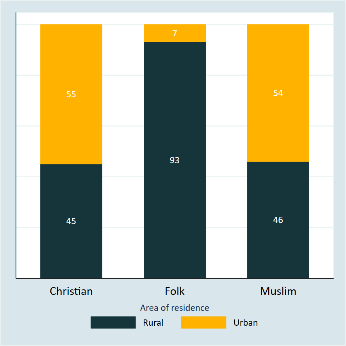** |
| **Uganda**  **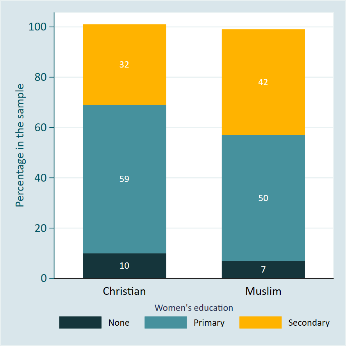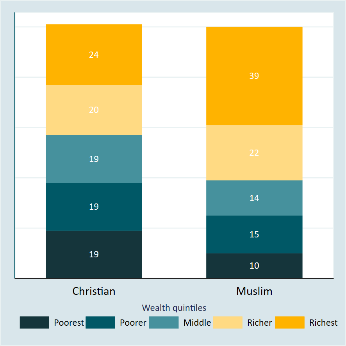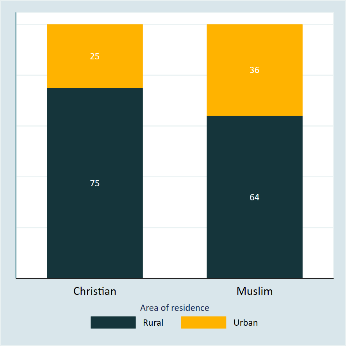** |  |

**Supplementary figure S2 – Crude and adjusted prevalence ratios (Muslim/Christian) for (a) full immunization coverage and (b) unvaccinated children by religious group, sorted by proportion of Christians in the population.**

**Supplementary table S3 – Crude and adjusted prevalence ratios from Poisson regression models for full immunization coverage, BCG, Polio, Measles, DPT, and percentage of unvaccinated children, by country.**

| **Country** | **Full immunization** | |  | **BCG** | |  | **Polio** | |  | **Measles** | |  | **DPT** | |  | **Unvaccinated** | |
| --- | --- | --- | --- | --- | --- | --- | --- | --- | --- | --- | --- | --- | --- | --- | --- | --- | --- |
|  | **Crude** | **Adjusted #** |  | **Crude** | **Adjusted #** |  | **Crude** | **Adjusted #** |  | **Crude** | **Adjusted #** |  | **Crude** | **Adjusted #** |  | **Crude** | **Adjusted #** |
| Benin | 0.81 a | 0.85 a |  | 0.87 a | 0.90 a |  | 0.85 a | 0.88 a |  | 0.85 a | 0.90 a |  | 0.84 a | 0.88 a |  | 3.69 b | 2.46 b |
| Burkina Faso | 0.95 | 0.94 a |  | 0.99 | 0.98 |  | 1.00 | 0.99 |  | 0.96 a | 0.95 a |  | 0.96 | 0.95 a |  | 0.75 | 0.81 |
| Cameroon | 0.86 a | 0.94 |  | 0.93 a | 0.98 |  | 0.99 | 1.03 |  | 0.85 a | 0.91 |  | 0.83 a | 0.91 |  | 0.81 | 0.35 b |
| Chad | 0.36 a | 0.37 a |  | 0.62 a | 0.65 a |  | 0.45 a | 0.46 a |  | 0.60 a | 0.64 a |  | 0.44 a | 0.43 a |  | 6.29 b | 4.76 b |
| Côte d’Ivoire | 0.72 a | 0.79 a |  | 0.89 a | 0.89 a |  | 0.88 a | 0.89 a |  | 0.78 a | 0.84 a |  | 0.79 a | 0.84 a |  | 2.18 b | 2.02 b |
| Ethiopia | 0.55 a | 0.63 a |  | 0.79 a | 0.84 a |  | 0.71 a | 0.77 a |  | 0.70 a | 0.76 a |  | 0.67 a | 0.75 a |  | 1.87 b | 1.56 b |
| Ghana | 1.02 | 1.04 |  | 0.99 | 1.01 |  | 1.06 | 1.08 b |  | 0.96 | 0.99 |  | 1.04 | 1.07 b |  | 1.03 | 0.54 |
| Guinea-Bissau | 0.91 a | 0.94 |  | 0.97 | 0.98 |  | 0.94 | 0.94 |  | 0.93 | 0.95 |  | 0.93 a | 0.95 |  | 9.78 b | 9.16 b |
| Liberia | 1.31 b | 1.31 b |  | 1.04 | 1.04 b |  | 1.16 | 1.17 b |  | 1.19 b | 1.21 b |  | 1.28 b | 1.28 b |  | 0.22b | 0.23 |
| Malawi | 0.94 | 0.95 |  | 0.99 | 1.00 |  | 0.96 | 0.96 |  | 0.96 a | 0.97 |  | 1.00 | 1.01 |  | 0.93 | 0.77 |
| Mozambique | 1.20 b | 1.27 b |  | 1.03 | 1.03 |  | 1.18 b | 1.23 b |  | 1.06 | 1.08 b |  | 1.16 b | 1.20 b |  | 0.80 | 0.77 |
| Nigeria | 0.38 a | 0.76 a |  | 0.53 a | 0.79 a |  | 0.58 a | 0.84 a |  | 0.47 a | 0.75 a |  | 0.38 a | 0.69 a |  | 3.00 b | 1.56 b |
| Sierra Leone | 0.91 a | 0.91 a |  | 0.99 | 0.99 |  | 0.95 | 0.95 |  | 0.94 | 0.95 |  | 0.96 | 0.96 |  | 1.74 | 1.57 |
| Togo | 1.10 | 1.15 b |  | 0.99 | 1.00 |  | 1.02 | 1.02 |  | 1.07 | 1.11 b |  | 0.99 | 1.01 |  | 1.41 | 1.20 |
| Uganda | 0.82 a | 0.83 a |  | 0.98 | 0.98 |  | 0.84 a | 0.86 a |  | 0.89 a | 0.87 a |  | 0.93 a | 0.93 |  | 1.92 | 2.46 b |

^#^ Full model adjusted for women’s education, wealth quintiles, and urban/rural residence

^a^ Higher prevalence among Christians (p value <0.05 based on crude Poisson regression models)

^b^ Higher prevalence among Muslims (p <0.05)

**Supplementary table S4 – Crude and adjusted ratios from Poisson regression models for full immunization coverage and percentage of unvaccinated children according to religious affiliation.**

| **Country** | **Religion** | **Unadjusted** | | | | **Women’s education** | | | | **Wealth quintiles** | | | | **Urban/rural residence** | | | | **Full adjustment^#^** | | | |
| --- | --- | --- | --- | --- | --- | --- | --- | --- | --- | --- | --- | --- | --- | --- | --- | --- | --- | --- | --- | --- | --- |
|  |  | **Reference: Christian** | | **Reference: Muslim** | | **Reference: Christian** | | **Reference: Muslim** | | **Reference: Christian** | | **Reference: Muslim** | | **Reference: Christian** | | **Reference: Muslim** | | **Reference: Christian** | | **Reference: Muslim** | |
|  |  | **PR** | **p value** | **PR** | **p value** | **PR** | **p value** | **PR** | **p value** | **PR** | **p value** | **PR** | **p value** | **PR** | **p value** | **PR** | **p value** | **PR** | **p value** | **PR** | **p value** |
| **Full immunization coverage** | | | | | | | | | | | | | | | | | | | | | |
| Benin | Muslim | 0.82^a^ | 0.00 | ×× | ×× | 0.84 ^a^ | 0.014 | ×× | ×× | 0.86 ^a^ | 0.02 | ×× | ×× | 0.82 ^a^ | 0.00 | ×× | ×× | 0.86 ^a^ | 0.02 | ×× | ×× |
|  | Folk | 0.91 | 0.21 | 1.11 | 0.231 | 0.96 | 0.55 | 1.13 | 0.175 | 1.02 | 0.75 | 1.18 | 0.052 | 0.92 | 0.25 | 1.11 | 0.215 | 1.03 | 0.72 | 1.19^c^ | 0.046 |
| Burkina Faso | Muslim | 0.95 | 0.06 | ×× | ×× | 0.96 | 0.07 | ×× | ×× | 0.95 | 0.06 | ×× | ×× | 0.95 ^a^ | 0.03 | ×× | ×× | 0.94 ^a^ | 0.03 | ×× | ×× |
| Cameroon | Muslim | 0.86 ^a^ | 0.01 | ×× | ×× | 0.97 | 0.57 | ×× | ×× | 0.87 ^a^ | 0.02 | ×× | ×× | 0.9 | 0.06 | ×× | ×× | 0.95 | 0.32 | ×× | ×× |
| Chad | Muslim | 0.36 ^a^ | <0.0001 | ×× | ×× | 0.41 ^a^ | <0.0001 | ×× | ×× | 0.35 ^a^ | <0.0001 | ×× | ×× | 0.34 ^a^ | <0.0001 | ×× | ×× | 0.37 ^a^ | <0.0001 | ×× | ×× |
| Côte d’Ivoire | Muslim | 0.70 ^a^ | <0.0001 | ×× | ×× | 0.79 ^a^ | 0.01 | ×× | ×× | 0.69 ^a^ | <0.0001 | ×× | ×× | 0.72 ^a^ | <0.0001 | ×× | ×× | 0.80 ^a^ | 0.01 | ×× | ×× |
| Ethiopia | Muslim | 0.55 ^a^ | <0.0001 | ×× | ×× | 0.60 ^a^ | <0.0001 | ×× | ×× | 0.57 ^a^ | <0.0001 | ×× | ×× | 0.61 ^a^ | <0.0001 | ×× | ×× | 0.63 ^a^ | <0.0001 | ×× | ×× |
| Ghana | Muslim | 1.02 | 0.66 | ×× | ×× | 1.04 | 0.51 | ×× | ×× | 1.02 | 0.66 | ×× | ×× | 1.02 | 0.74 | ×× | ×× | 1.04 | 0.49 | ×× | ×× |
| Guinea Bissau | Muslim | 0.90 ^a^ | 0.03 | ×× | ×× | 0.98 | 0.73 | ×× | ×× | 0.88 ^a^ | 0.01 | ×× | ×× | 0.91 ^a^ | 0.05 | ×× | ×× | 0.94 | 0.31 | ×× | ×× |
|  | Folk | 0.89 | 0.12 | 0.99 | 0.882 | 0.96 | 0.59 | 0.98 | 0.759 | 0.96 | 0.52 | 1.09 | 0.253 | 0.91 | 0.15 | 0.99 | 0.969 | 0.98 | 0.83 | 1.04 | 0.56 |
| Liberia | Muslim | 1.31 ^b^ | 0.00 | ×× | ×× | 1.34 ^b^ | 0.00 | ×× | ×× | 1.32 ^b^ | 0.00 | ×× | ×× | 1.26 ^b^ | 0.01 | ×× | ×× | 1.31 ^b^ | 0.00 | ×× | ×× |
| Malawi | Muslim | 0.94 | 0.07 | ×× | ×× | 0.95 | 0.14 | ×× | ×× | 0.94 | 0.06 | ×× | ×× | 0.94 | 0.09 | ×× | ×× | 0.95 | 0.15 | ×× | ×× |
| Mozambique | Muslim | 1.20 ^b^ | 0.01 | ×× | ×× | 1.25 ^b^ | 0.00 | ×× | ×× | 1.23 ^b^ | 0.00 | ×× | ×× | 0.95 | 0.44 | ×× | ×× | 1.27 ^b^ | <0.0001 | ×× | ×× |
|  | Folk | 1.06 | 0.43 | 0.88 | 0.133 | 1.1 | 0.21 | 0.88 | 0.111 | 1.03 | 0.67 | 0.83^b^ | 0.042 | 1.17 ^c^ | 0.01 | 0.91 | 0.262 | 1.03 | 0.63 | 0.81^b^ | 0.015 |
| Nigeria | Muslim | 0.37 ^a^ | <0.0001 | ×× | ×× | 0.75 ^a^ | 0.00 | ×× | ×× | 0.41 ^a^ | <0.0001 | ×× | ×× | 0.57 ^a^ | <0.0001 | ×× | ×× | 0.73 ^a^ | <0.0001 | ×× | ×× |
| Sierra Leone | Muslim | 0.91 ^a^ | 0.04 | ×× | ×× | 0.92 | 0.09 | ×× | ×× | 0.90 ^a^ | 0.03 | ×× | ×× | 0.89 ^a^ | 0.02 | ×× | ×× | 0.91 ^a^ | 0.04 | ×× | ×× |
| Togo | Muslim | 1.1 | 0.08 | ×× | ×× | 1.16 ^b^ | 0.01 | ×× | ×× | 1.09 | 0.12 | ×× | ×× | 1.09 | 0.09 | ×× | ×× | 1.15 ^b^ | 0.02 | ×× | ×× |
|  | Folk | 0.77 ^a^ | 0.01 | 0.70 ^b^ | 0.001 | 0.86 | 0.10 | 0.74 ^b^ | 0.003 | 0.78 ^a^ | 0.01 | 0.72 ^b^ | 0.001 | 0.79 ^a^ | 0.01 | 0.72 ^b^ | 0.002 | 0.85 | 0.06 | 0.73 ^b^ | 0.002 |
| Uganda | Muslim | 0.82 ^a^ | 0.01 | ×× | ×× | 0.82 ^a^ | 0.00 | ×× | ×× | 0.82 ^a^ | 0.01 | ×× | ×× | 0.82 ^a^ | 0.01 | ×× | ×× | 0.83 ^a^ | 0.01 | ×× | ×× |
| **Unvaccinated children** | | | | | | | | | | | | | | | | | | | | | |
| Benin | Muslim | 3.69 | 0.00 | ×× | ×× | 2.95 | 0.00 | ×× | ×× | 2.68 | 0.00 | ×× | ×× | 3.58 | 0.00 | ×× | ×× | 2.46 | 0.00 | ×× | ×× |
|  | Folk | 2.05 | 0.01 | 0.55 | 0.00 | 1.63 | 0.07 | 0.55 | 0.02 | 1.20 | 0.50 | 0.45 | 0.00 | 1.95 | 0.02 | 0.54 | 0.02 | 1.14 | 0.64 | 0.46 | 0.00 |
| Burkina Faso | Muslim | 0.75 | 0.53 | ×× | ×× | 0.66 | 0.37 | ×× | ×× | 0.89 | 0.80 | ×× | ×× | 0.74 | 0.53 | ×× | ×× | 0.81 | 0.66 | ×× | ×× |
| Cameroon | Muslim | 0.81 | 0.67 | ×× | ×× | 0.33 | 0.05 | ×× | ×× | 0.59 | 0.31 | ×× | ×× | 0.75 | 0.57 | ×× | ×× | 0.35 | 0.06 | ×× | ×× |
| Chad | Muslim | 6.29 | 0.00 | ×× | ×× | 4.14 | 0.00 | ×× | ×× | 6.83 | 0.00 | ×× | ×× | 6.74 | 0.00 | ×× | ×× | 4.76 | 0.00 | ×× | ×× |
| Côte d’Ivoire | Muslim | 2.18 | 0.00 | ×× | ×× | 1.75 | 0.02 | ×× | ×× | 2.16 | 0.00 | ×× | ×× | 2.33 | 0.00 | ×× | ×× | 2.02 | 0.00 | ×× | ×× |
| Ethiopia | Muslim | 1.87 | 0.00 | ×× | ×× | 1.74 | 0.00 | ×× | ×× | 1.61 | 0.01 | ×× | ×× | 1.76 | 0.00 | ×× | ×× | 1.56 | 0.01 | ×× | ×× |
| Ghana | Muslim | 1.03 | 0.98 | ×× | ×× | 0.57 | 0.51 | ×× | ×× | 0.84 | 0.84 | ×× | ×× | 1.03 | 0.98 | ×× | ×× | 0.54 | 0.45 | ×× | ×× |
| Guinea Bissau | Muslim | 9.78 | 0.03 | ×× | ×× | 6.24 | 0.03 | ×× | ×× | 11.08 | 0.02 | ×× | ×× | 7.16 | 0.05 | ×× | ×× | 9.16 | 0.02 | ×× | ×× |
|  | Folk | 5.54 | 0.11 | 0.57 | 0.03 | 3.80 | 0.14 | 0.61 | 0.22 | 2.64 | 0.33 | 0.24 | 0.00 | 3.30 | 0.24 | 0.46 | 0.05 | 2.37 | 0.35 | 0.26 | 0.00 |
| Liberia | Muslim | 0.22 | 0.15 | ×× | ×× | 0.17 | 0.10 | ×× | ×× | 0.26 | 0.20 | ×× | ×× | 0.21 | 0.13 | ×× | ×× | 0.23 | 0.14 | ×× | ×× |
| Malawi | Muslim | 0.93 | 0.88 | ×× | ×× | 0.77 | 0.58 | ×× | ×× | 0.92 | 0.85 | ×× | ×× | 0.93 | 0.88 | ×× | ×× | 0.77 | 0.58 | ×× | ×× |
| Mozambique | Muslim | 0.80 | 0.63 | ×× | ×× | 0.76 | 0.59 | ×× | ×× | 0.74 | 0.54 | ×× | ×× | 0.82 | 0.69 | ×× | ×× | 0.77 | 0.61 | ×× | ×× |
|  | Folk | 1.73 | 0.25 | 2.17 | 0.23 | 1.66 | 0.29 | 2.18 | 0.23 | 2.27 | 0.06 | 3.08 | 0.11 | 1.60 | 0.33 | 1.94 | 0.31 | 2.47 | 0.05 | 3.22 | 0.08 |
| Nigeria | Muslim | 3.00 | 0.00 | ×× | ×× | 1.55 | 0.00 | ×× | ×× | 2.10 | 0.00 | ×× | ×× | 2.78 | 0.00 | ×× | ×× | 1.56 | 0.00 | ×× | ×× |
| Sierra Leone | Muslim | 1.74 | 0.14 | ×× | ×× | 1.49 | 0.27 | ×× | ×× | 1.86 | 0.10 | ×× | ×× | 1.79 | 0.13 | ×× | ×× | 1.57 | 0.21 | ×× | ×× |
| Togo | Muslim | 1.41 | 0.48 | ×× | ×× | 1.17 | 0.73 | ×× | ×× | 1.47 | 0.43 | ×× | ×× | 1.42 | 0.47 | ×× | ×× | 1.20 | 0.70 | ×× | ×× |
|  | Folk | 5.04 | 0.00 | 3.58 | 0.00 | 3.60 | 0.00 | 3.09 | 0.01 | 4.88 | 0.00 | 3.31 | 0.01 | 4.50 | 0.00 | 3.17 | 0.02 | 3.84 | 0.00 | 3.20 | 0.02 |
| Uganda | Muslim | 1.92 | 0.13 | ×× | ×× | 2.16 | 0.07 | ×× | ×× | 2.30 | 0.06 | ×× | ×× | 2.21 | 0.07 | ×× | ×× | 2.46 | 0.04 | ×× | ×× |

^#^ Full model adjusted for women’s education, wealth quintiles, and urban/rural residence

**Supplementary table S5 – Poisson regression coefficients for interactions between sex of the child and religion for full immunization coverage and non-vaccinated children**.

| **Country** | **Unadjusted** | | | | | | | |  | **Full adjustment^#^** | | | | | | | |
| --- | --- | --- | --- | --- | --- | --- | --- | --- | --- | --- | --- | --- | --- | --- | --- | --- | --- |
|  | **PR sex** | **p value** |  | **PR religion** | **p value** | **PR interaction** | **p value** | **model p value** |  | **PR sex** | **p value** |  | **PR religion** | **p value** | **PR interaction** | **p value** | **model p value** |
| **Full immunization** |  |  |  |  |  |  |  |  |  |  |  |  |  |  |  |  |  |
| Benin | 0.97 | 0.583 | Muslim | 0.82 | 0.023** | 0.98 | 0.816 | 0.052 |  | 0.98 | 0.68 | Muslim | 0.86 | 0.088 | 0.99 | 0.949 | <0.001 |
|  |  |  | Folk | 0.88 | 0.191 | 1.07 | 0.565 |  |  |  |  | Folk | 0.99 | 0.924 | 1.07 | 0.594 |  |
| Burkina Faso | 0.94 | 0.053 | Muslim | 0.93 | 0.023** | 1.06 | 0.224 | 0.047 |  | 0.94 | 0.06 | Muslim | 0.92 | 0.009 | 1.06 | 0.205 | 0.031 |
| Cameroon | 1.13 | 0.001** | Muslim | 0.95 | 0.494 | 0.8 | 0.02** | 0.000 |  | 1.14 | <0.0001 | Muslim | 1.05 | 0.378 | 0.79 | 0.022 | <0.001 |
| Chad | 0.99 | 0.920 | Muslim | 0.33 | <0.001** | 1.19 | 0.339 | <0.001** |  | 1.02 | 0.84 | Muslim | 0.35 | <0.0001 | 1.12 | 0.517 | <0.001 |
| Côte d'Ivoire | 0.95 | 0.571 | Muslim | 0.66 | <0.001** | 1.12 | 0.400 | <0.001** |  | 0.98 | 0.87 | Muslim | 0.75 | 0.010 | 1.13 | 0.375 | <0.001 |
| Ethiopia | 1.08 | 0.368 | Muslim | 0.54 | 0.001** | 1.02 | 0.913 | 0.001** |  | 1.06 | 0.51 | Muslim | 0.62 | 0.008 | 1.01 | 0.953 | <0.001 |
| Ghana | 0.98 | 0.581 | Muslim | 1.07 | 0.187 | 0.91 | 0.261 | 0.314 |  | 0.98 | 0.56 | Muslim | 1.09 | 0.133 | 0.90 | 0.251 | 0.785 |
| Guinea-Bissau | 0.93 | 0.371 | Muslim | 0.87 | 0.020** | 1.08 | 0.375 | 0.151 |  | 0.95 | 0.55 | Muslim | 0.92 | 0.220 | 1.06 | 0.508 | <0.001 |
|  |  |  | Folk | 0.83 | 0.039** | 1.17 | 0.186 |  |  |  |  | Folk | 0.93 | 0.448 | 1.12 | 0.339 |  |
| Liberia | 1.12 | 0.169 | Muslim | 1.43 | 0.003** | 0.84 | 0.310 | 0.014 |  | 1.11 | 0.20 | Muslim | 1.39 | 0.002 | 0.88 | 0.460 | <0.001 |
| Malawi | 1.02 | 0.555 | Muslim | 0.93 | 0.177 | 1.02 | 0.793 | 0.297 |  | 1.01 | 0.57 | Muslim | 0.94 | 0.278 | 1.01 | 0.863 | <0.001 |
| Mozambique | 0.88 | 0.149 | Muslim | 1.12 | 0.221 | 1.16 | 0.244 | 0.097 |  | 0.89 | 0.15 | Muslim | 1.21 | 0.041 | 1.11 | 0.379 | <0.001 |
|  |  |  | Folk | 1.01 | 0.925 | 1.11 | 0.507 |  |  |  |  | Folk | 0.97 | 0.781 | 1.13 | 0.395 |  |
| Nigeria | 1.00 | 0.956 | Muslim | 0.37 | <0.001** | 1.03 | 0.840 | <0.001 |  | 1.06 | 0.39 | Muslim | 0.72 | 0.001 | 1.02 | 0.912 | <0.001 |
| Sierra Leone | 1.03 | 0.671 | Muslim | 0.93 | 0.218 | 0.97 | 0.688 | 0.213 |  | 1.04 | 0.60 | Muslim | 0.93 | 0.260 | 0.96 | 0.616 | 0.007 |
| Togo | 0.92 | 0.177 | Muslim | 1.08 | 0.254 | 1.02 | 0.840 | 0.023 |  | 0.93 | 0.22 | Muslim | 1.14 | 0.067 | 1.00 | 0.966 | 0.003 |
|  |  |  | Folk | 0.73 | 0.006** | 1.11 | 0.407 |  |  |  |  | Folk | 0.81 | 0.060 | 1.07 | 0.563 |  |
| Uganda | 0.96 | 0.303 | Muslim | 0.83 | 0.037** | 0.97 | 0.842 | 0.020 |  | 0.97 | 0.34 | Muslim | 0.84 | 0.047 | 0.97 | 0.828 | 0.078 |
| **Unvaccinated children** | |  |  |  |  |  |  |  |  |  |  |  |  |  |  |  |  |
| Benin | 1.38 | 0.283 | Muslim | 4.55 | 0.000 | 0.67 | 0.261 | 0.000 |  | 1.40 | 0.25 | Muslim | 3.18 | 0.000 | 0.61 | 0.140 | 0.000 |
|  |  |  | Folk | 2.25 | 0.036 | 0.83 | 0.741 |  |  |  |  | Folk | 1.26 | 0.546 | 0.81 | 0.709 |  |
| Burkina Faso ± |  |  | Muslim |  |  |  |  |  |  |  |  | Muslim |  |  |  |  |  |
| Cameroon | 1.01 | 0.990 | Muslim | 0.72 | 0.622 | 1.26 | 0.809 | 0.954 |  | 0.97 | 0.10 | Muslim | 0.31 | 0.102 | 1.28 | 0.794 | 0.000 |
| Chad | 1.26 | 0.367 | Muslim | 6.78 | 0.000 | 0.87 | 0.592 | 0.000 |  | 1.23 | 0.41 | Muslim | 5.00 | 0.000 | 0.91 | 0.735 | 0.000 |
| Côte d'Ivoire | 0.50 | 0.072 | Muslim | 1.53 | 0.158 | 2.30 | 0.062 | 0.002 |  | 0.47 | 0.06 | Muslim | 1.42 | 0.276 | 2.30 | 0.065 | 0.000 |
| Ethiopia | 1.15 | 0.526 | Muslim | 2.13 | 0.001 | 0.79 | 0.439 | 0.005 |  | 1.15 | 0.51 | Muslim | 1.77 | 0.012 | 0.79 | 0.509 | 0.000 |
| Ghana | 0.98 | 0.977 | Muslim | 1.79 | 0.559 | 0.14 | 0.177 | 0.486 |  | 0.96 | 0.95 | Muslim | 0.97 | 0.980 | 0.13 | 0.154 | 0.004 |
| Guinea-Bissau ± |  |  | Muslim |  |  |  |  |  |  |  |  | Muslim |  |  |  |  |  |
|  |  |  | Folk |  |  |  |  |  |  |  |  | Folk |  |  |  |  |  |
| Liberia ± |  |  | Muslim |  |  |  |  |  |  |  |  | Muslim |  |  |  |  |  |
| Malawi | 1.62 | 0.192 | Muslim | 2.04 | 0.192 | 0.15 | 0.109 | 0.331 |  | 1.63 | 0.18 | Muslim | 1.70 | 0.332 | 0.15 | 0.110 | 0.322 |
| Mozambique | 1.45 | 0.432 | Muslim | 1.59 | 0.418 | 0.16 | 0.069 | 0.403 |  | 1.42 | 0.45 | Muslim | 1.38 | 0.609 | 0.20 | 0.112 | 0.000 |
|  |  |  | Folk | 2.08 | 0.263 | 0.71 | 0.629 |  |  |  |  | Folk | 3.15 | 0.057 | 0.64 | 0.485 |  |
| Nigeria | 0.90 | 0.358 | Muslim | 2.81 | 0.000 | 1.14 | 0.301 | 0.000 |  | 0.86 | 0.18 | Muslim | 1.47 | 0.000 | 1.12 | 0.344 | 0.000 |
| Sierra Leone | 0.94 | 0.933 | Muslim | 1.85 | 0.311 | 0.88 | 0.887 | 0.501 |  | 0.87 | 0.86 | Muslim | 1.58 | 0.441 | 0.99 | 0.991 | 0.009 |
| Togo | 0.36 | 0.111 | Muslim | 0.76 | 0.668 | 4.31 | 0.129 | 0.006 |  | 0.34 | 0.09 | Muslim | 0.61 | 0.410 | 4.92 | 0.091 | 0.002 |
|  |  |  | Folk | 4.19 | 0.003 | 1.69 | 0.539 |  |  |  |  | Folk | 2.89 | 0.018 | 1.93 | 0.447 |  |
| Uganda | 1.67 | 0.244 | Muslim | 2.88 | 0.052 | 0.45 | 0.374 | 0.245 |  | 1.69 | 0.23 | Muslim | 3.83 | 0.015 | 0.41 | 0.324 | 0.000 |

PR = Prevalence ratio tested using Poisson regression models

NA = Not available

References: Male and Christian categories

^#^ Full model adjusted for women’s education, wealth quintiles, and urban/rural residence

* p value < 0.10; ** p value < 0.05

± Prevalence equal to zero for one of the categories

**Supplementary table S6 – Pearson’s coefficients of correlation between crude and adjusted ratios (Muslim/Christian) of full immunization coverage and proportion of unvaccinated children.**

|  | **Full immunization coverage** | | | | **Unvaccinated children** | | | |
| --- | --- | --- | --- | --- | --- | --- | --- | --- |
|  | **Crude ratio** | | **Fully adjusted ratio^#^** | | **Crude ratio** | | **Fully adjusted ratio^#^** | |
|  | **Pearson’s r** | **p value** | **Pearson’s r** | **p value** | **Pearson’s r** | **p value** | **Pearson’s r** | **p value** |
| **National coverage** | 0.726 | <0.01* | 0.610 | 0.016* | -0.018 | 0.948 | -0.142 | 0.614 |
| **Muslim (%)** | -0.485 | 0.067 | -0.467 | 0.079 | 0.358 | 0.189 | 0.313 | 0.257 |
| **Christian (%)** | 0.339 | 0.217 | 0.316 | 0.251 | -0.471 | 0.077 | -0.432 | 0.108 |

^#^ Full adjustment for women’s education, wealth quintiles, and urban/rural residence
